# Improving Short‐ and Long‐Term Type 2 Diabetes Risk Prediction for Middle‐Aged British Adults

**DOI:** 10.1111/dom.71289

**Published:** 2026-08-26

**Authors:** Cunrong Huang, Andre Lopes, Annie Britton

**Affiliations:** ^1^ Department of Epidemiology and Public Health University College London London UK; ^2^ Cancer Research UK and Cancer Trials Centre University College London London UK

**Keywords:** general practice, long‐term risk, prediction tool, short‐term risk, type 2 diabetes

## Abstract

**Aims:**

Diabetes prediction tools are valuable for prevention and resource planning. Few tools exist to predict long‐term diabetes risk for British adults. We aim to develop and validate a diabetes prediction tool for short‐ and long‐term risk.

**Materials and Methods:**

Prediction model development (Training, *n* = 4502) and internal validation (Test, *n* = 1929) used Whitehall II data (WHII); external validation used English Longitudinal Study of Ageing (ELSA) data, with incident diabetes as outcome. Predictors were selected using LASSO‐Cox, random survival forests (RSF) and best subset selection. Three candidate Cox models were built from these predictor sets and compared. Performance was assessed with *C*‐indices, calibration and decision curve analysis (DCA). A continuous risk score and quartile‐based risk groups were derived; a nomogram was created.

**Results:**

Across WHII Test (235 events) and ELSA (255 events), three models showed broadly similar discrimination, calibration and DCA patterns. LASSO‐ and RSF‐selected models performed comparably and slightly better than best subset. LASSO and RSF both selected ethnicity, body mass index, waist‐to‐height ratio, height, triglyceride‐glucose index, fasting glucose, family history of diabetes and socioeconomic status; LASSO additionally selected smoking, whereas RSF also included age, triglycerides, high‐density lipoprotein cholesterol, systolic and diastolic blood pressure. In ELSA, risk‐score *C*‐indices (95% CIs) were 0.800 (0.773–0.827) for the LASSO‐selected model and 0.801 (0.774–0.827) for the RSF‐selected model; risk‐group *C*‐indices were 0.737 (0.715–0.759) and 0.727 (0.707–0.748), respectively.

**Conclusions:**

We developed and externally validated a tool to estimate 5‐, 10‐ and 15‐year diabetes risk in British middle‐aged adults, which may support primary care decision‐making.

## 1 Introduction

Although cardiovascular disease (CVD) remains the leading cause of death worldwide, significant progress has been made in its prevention and treatment over the past three to four decades [1, 2]. For CVD prevention, the Framingham Risk Score may have contributed to these advances by estimating an individual's 10‐year cardiovascular risk and enabling early identification of individuals at higher risk [3, 4, 5]. Unlike CVD rates, the prevalence of Type 2 diabetes has been rising continuously in recent years [6]. Similar to the Framingham Risk Score for predicting CVD risk, several tools exist for diabetes risk prediction [7, 8, 9, 10, 11, 12, 13, 14]. However, these tools have not been widely used in general practice. The use of the Framingham Risk Score in CVD prevention suggests the potential of prediction models for preventing and reducing disease incidence. There remains room for improvement in diabetes risk prediction.

Obesity is a key modifiable risk factor for diabetes [1, 15]. Most existing prediction models use body mass index (BMI) as a predictor, while waist‐based measures, another index for assessing obesity, are less frequently considered. When waist‐based measures are included, waist circumference (WC) is typically the only one used. Some studies have suggested that waist‐to‐height ratio (WHtR), a waist‐based measure, is as strongly associated with diabetes risk as WC but is less influenced by sex and ethnicity [16, 17, 18, 19, 20]. However, its role in prediction models has been less well established. Therefore, the potential value of incorporating WHtR in addition to commonly used measures warrants further evaluation.

Moreover, most existing prediction models estimate risk at a single prediction horizon, such as a 5‐year or 10‐year risk [7, 21]. However, for middle‐aged (40+ years) adults, considering current average life expectancy, a 10‐year or shorter risk prediction may be insufficient, particularly since diabetes risk may progressively increase with age [22, 23]. Therefore, using long‐term prospective data, this study aims to develop a tool to predict individual risk at 5, 10 and 15 years and to support prevention planning and health management across shorter and longer time horizons. In addition to quantitative risk estimates, the present tool provides categorical risk stratification, which may support risk communication, monitoring and use in different practice settings. Some existing tools provide only one form of risk information, which may limit risk assessment in practice. Additionally, the present study applies complementary statistical approaches to identify key predictors from routinely available variables and to develop a parsimonious prediction tool.

Overall, this study aims to develop and externally validate a parsimonious diabetes risk prediction tool based on routinely available predictors for use in British general practice.

## 2 Materials and Methods

### 2.1 Data Collection

This study used data from Whitehall II (WHII) cohort study to build the prediction model for diabetes risk and conduct internal validation, and data from English longitudinal study of ageing (ELSA) for external validation [24, 25, 26]. Descriptions of both cohorts are summarised in Table S1. ELSA was selected as an independent, population‐based cohort in England to evaluate the prediction model beyond the occupational WHII cohort. Ethical approval was obtained for both WHII and ELSA, and all participants provided informed consent.

### 2.2 Study Sample

In WHII, participants without baseline diabetes and with complete Phase 3 (1991–1994) data on all candidate predictors (see Section 2.3) were included in the primary complete‐case analysis and followed until Phase 9 (2007–2009). To internally validate the prediction model, we randomly split the included participants into a Training set (70%) for model training and a Test set (30%) for validation [27]. A flow chart of the included participants is shown in Figure 1. Missing candidate predictor data before complete‐case exclusion are reported in Table S2. In ELSA (external validation), we included persons without diabetes at baseline who had complete data for the key variables of the candidate prediction models. The candidate models were developed using different variable screening methods (see Section 2.5.1) in the WHII Training set. External validation in ELSA was performed using data from Wave 2 (2004–2005), with follow‐up until Wave 10 (2021–2023).

**FIGURE 1:**
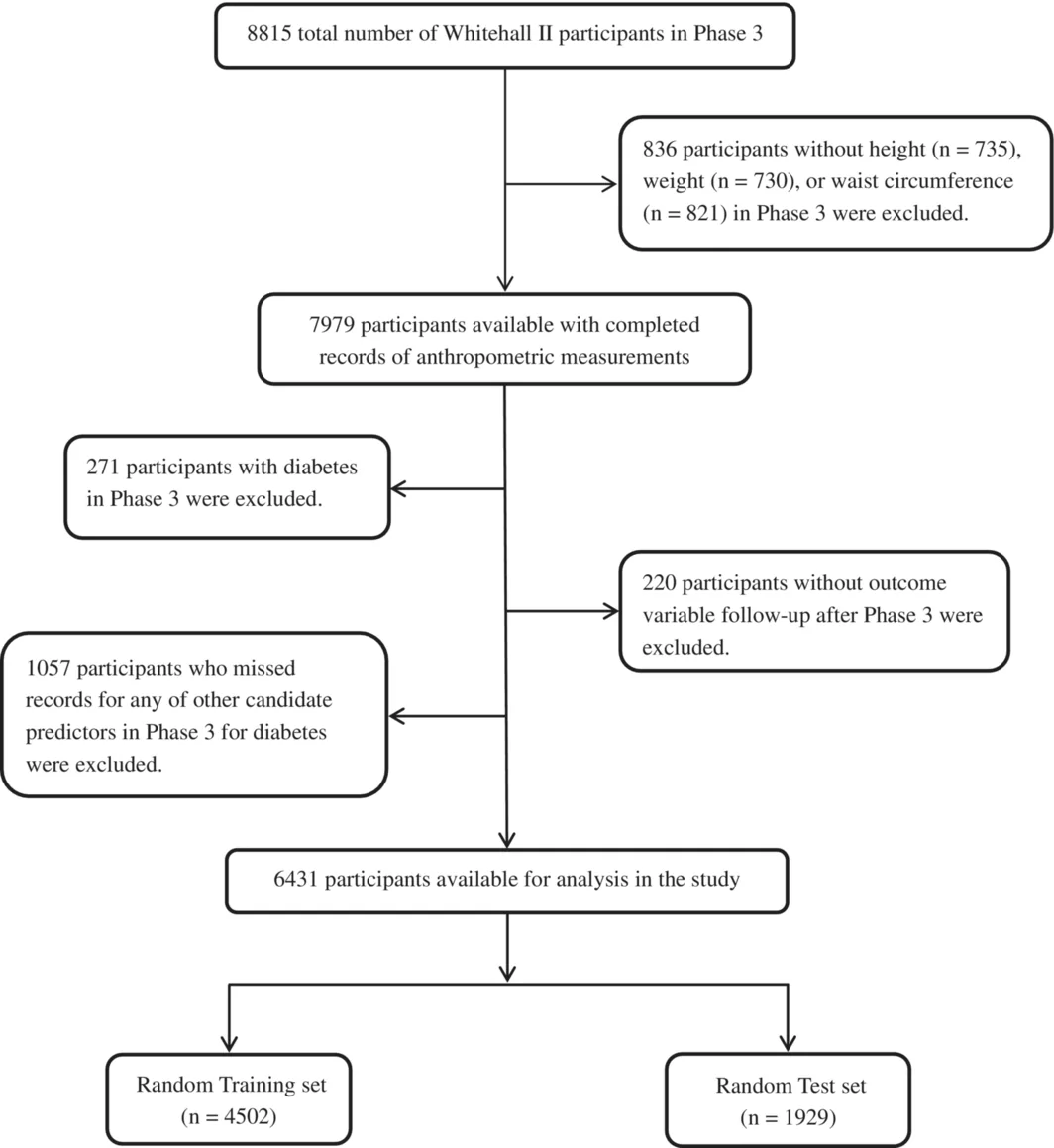
Flow chart of the included participants in Whitehall II.

### 2.3 Candidate Predictors (Initial Variable Pool: 21 Variables in WHII Training Set)

The candidate predictors included age (continuous, years), sex (male or female), ethnicity (white or non‐white), height (continuous, m), BMI (continuous, weight[kg]/height[m]^2^), WHtR (continuous, WC[cm]/height[cm]), systolic blood pressure (SBP, continuous, mmHg), diastolic blood pressure (DBP, continuous, mmHg), triglycerides (TG, continuous, mmol/L), low‐density lipoprotein cholesterol (LDL‐C, continuous, mmol/L), high‐density lipoprotein cholesterol (HDL‐C, continuous, mmol/L), triglyceride‐glucose index (TyG index, continuous, TyG index = ln(fasting triglycerides [mg/dL] × fasting plasma glucose [mg/dL]/2)), fasting glucose (continuous, mmol/L), family history of diabetes (yes or no), smoking (never smoker, ex‐smoker, current smoker), drinking (non‐current, current moderate, current heavy), fruit/vegetable consumption (< daily or ≥ daily), physical activity (inactive, moderate, or active), socioeconomic status (SES: low, intermediate and high), baseline CVD diagnosis (self‐reported physician‐diagnosed CVD; yes or no) and CVD medication (use of medications for cardiovascular disease; yes or no). Detailed definitions of drinking status, SES, physical activity, baseline CVD diagnosis and CVD medication are provided in Table S3. Candidate predictors were selected a priori based on prior literature, clinical relevance to diabetes and data availability [7, 9, 12, 13].

### 2.4 Outcome

Incident Type 2 diabetes was defined as having a fasting plasma glucose ≥ 7.0 mmol/L, a 2‐h postload glucose ≥ 11.1 mmol/L at clinical examination, participant report of physician‐diagnosed Type 2 diabetes, use of diabetes medication, or a hospital record of diabetes [28, 29]. Follow‐up began on the survey date of Phase 3 in WHII (Wave 2 in ELSA) and ended at the earliest occurrence of incident diabetes, death, emigration, the last attended follow‐up, or the administrative end of follow‐up (Phase 9 in WHII; Wave 10 in ELSA).

### 2.5 Statistical Analysis

#### 2.5.1 Analysis in WHII

##### 2.5.1.1 Selection of Key Predictors

Three methods, LASSO‐Cox regression, random survival forests (RSF) and best subset selection, were applied separately to select key predictors from the initial variable pool (full model, variables from WHII) for building a simplified model. These methods were chosen to represent complementary approaches to variable selection. LASSO uses penalisation to mitigate multicollinearity and reduce overfitting; RSF captures nonlinear relationships and interactions; best subset selection evaluates all possible predictor combinations and selects the subset that optimises model performance (see below). Using complementary methods reduces reliance on any single method and allows predictors to be evaluated from different methodological perspectives.

First, we used LASSO‐Cox regression with 10‐fold cross‐validation to identify predictors in the Training set. The penalty parameter (*λ*) was chosen as the largest value within one standard error of the minimum cross‐validated error [30, 31]. To ensure robustness of predictor selection, we performed LASSO‐Cox regression on 1000 bootstrap samples in the Training set. Variables appearing in ≥ 70% of samples were retained. Alternative thresholds (50%, 60%, 80%) were tested for sensitivity analysis. Second, we employed an RSF model to identify key predictors. Variable importance was calculated using permutation‐based measures to assess each variable's contribution to prediction accuracy. A random noise variable was used as a reference. Predictors with importance scores exceeding that of the noise variable were retained [32]. Third, we applied best subset selection to identify optimal predictors by evaluating all possible variable combinations, selecting the model that minimised the Akaike information criterion (AIC) and maximised predictive accuracy [33].

##### 2.5.1.2 Building the Prediction Model

Subsequently, three simplified Cox regression models were developed as candidate models, each using a different set of key predictors from different selection methods.

##### 2.5.1.3 Evaluation of Prediction Performance After Variable Selection

We evaluated discrimination (*C*‐index) and calibration of the three candidate models in the Training and WHII Test sets separately. Time‐dependent *C*‐indices were estimated with 1000 bootstraps. Bias‐corrected calibration curves based on 1000 bootstraps were compared with Kaplan–Meier estimates. Calibration slopes and 5‐, 10‐ and 15‐year observed‐to‐expected (O/E) ratios were also reported with 95% confidence intervals. These analyses provided internal validation and assessed whether the predictors selected by each method could support model discrimination and calibration.

##### 2.5.1.4 Comparison of Three Candidate Models

We then evaluated the performance of the candidate models in the WHII Test set and in the external ELSA using discrimination (time‐dependent *C*‐index) and decision curve analysis (DCA). Model complexity was considered when performance differences were minimal. DCA assessed clinical utility across the full probability range. As no universally accepted thresholds exist for diabetes risk intervention or screening across the prediction horizons examined, we followed a published approach and prespecified threshold probabilities of 25%, 50% and 75% to facilitate model comparison [34]. At each threshold, decision curves of the candidate models were compared with the default strategies of treating all or none. The optimal model at each follow‐up (5, 10, and 15 years) was defined as the one showing higher net benefit than the others and surpassing both default strategies.

##### 2.5.1.5 Developing the Prediction Tool

Candidate models selected based on an overall consideration of discrimination, calibration and DCA were then developed into prediction tools for use in general practice. As part of prediction‐tool development, the linearity of continuous predictors was assessed using restricted cubic splines, with details provided in Table S4. Multicollinearity was assessed using variance inflation factors (VIFs) in the WHII Training set. In the Training set, we fitted each selected Cox model to obtain coefficients for selected variables and the baseline survival function. The risk score was calculated as the sum of each regression coefficient multiplied by the observed value of each continuous variable or the coded indicator value of each categorical variable in the model. For presentation, coefficients were rounded to two decimals and multiplied by 100; higher scores indicated higher diabetes risk. Participants in the Training set were divided into quartiles of the risk score to form four relative risk groups (very low, low, medium, high) to ensure a balanced distribution of participants across groups.

##### 2.5.1.6 Validation of the Prediction Tool

Predicted risks from the Training‐set model were validated against observed diabetes incidence in the WHII Test set. Kaplan–Meier curves illustrated diabetes‐free probability across the four risk groups in the WHII Training set and the WHII Test set. Discriminatory ability of the risk score and risk‐group classification was assessed using the *C*‐index in the Training set and the WHII Test set. A nomogram was also constructed from the final simplified Cox model in the Training set: points were assigned to each predictor and summed to a total score to estimate individual predicted risk; higher total scores indicated greater predicted risk.

#### 2.5.2 Analysis in ELSA (External Validation)

In ELSA, *C*‐indices were calculated for candidate models, risk scores and risk groups and DCA was performed for candidate models. Training‐set predicted risks were compared with observed risks in ELSA and Kaplan–Meier curves were plotted across risk groups.

#### 2.5.3 Sensitivity Analysis

We examined whether prediction performance varied by the choice of adiposity index (BMI, waist‐based measure, or both) or by the inclusion of invasive variables (requiring a blood test). We conducted sensitivity analyses in the WHII Training set, testing (a) whether including WHtR significantly improved the *C*‐index and (b) whether including invasive variables significantly improved the *C*‐index [35]. As another sensitivity analysis, multiple imputation by chained equations (MICE) was applied to WHII data before complete‐case exclusion, generating 20 imputed datasets. The imputation model included all candidate predictors, follow‐up time and event status. The same predictor sets as in the primary analysis were used, and Cox regression coefficients for the predictors were pooled across the imputed datasets using Rubin's rules. The resulting continuous risk scores (as described in Section 2.5.1) were externally evaluated in ELSA using *C*‐indices with 95% confidence intervals.

#### 2.5.4 Additional Analysis Addressing Potential Selection‐Induced Optimism

To enhance transparency regarding potential selection‐induced optimism, an additional analysis was conducted for the candidate model not taken forward in the primary analysis. The prediction‐tool development procedures described above for deriving the risk score, risk groups and nomogram were applied.

All reported *p*‐values were two‐sided. All analyses were performed in R (version 4.4.1).

## 3 Results

### 3.1 Developing the Prediction Model

#### 3.1.1 Characteristics of Included Participants in WHII

A total of 6431 participants without diabetes at Phase 3 were included, with 4502 in the Training set and 1929 in the WHII Test set. The mean age was 49.95 years (SD: 6.01) and 1956 (30.4%) were female (Table 1). There were 782 incident diabetes cases recorded, with a median follow‐up of 16.1 years (Table S5). All candidate predictors in the initial variable pool were significantly associated with incident diabetes (Table S6).

**TABLE 1:** Baseline (Phase 3) characteristics of all participants in Whitehall II.

Characteristic	All, *N* (%)	WHII training set, *N* (%)	WHII test set, *N* (%)	*p* ^a^
	*N* = 6431 (100)	*N* = 4502 (70)	*N* = 1929 (30)	
Age, mean ± SD, years	49.95 ± 6.01	49.96 ± 6.00	49.91 ± 6.03	0.744
Range	(39.60, 63.10)	(39.60, 62.70)	(39.70, 63.10)	
Sex (Female), *N* (%)	1956 (30.4)	1397 (31.0)	559 (29.0)	0.108
Ethnicity (White), *N* (%)	5907 (91.9)	4143 (92.0)	1764 (91.4)	0.466
Height, mean ± SD, m	1.72 ± 0.09	1.72 ± 0.09	1.72 ± 0.09	0.950
BMI, mean ± SD, kg/m^2^	25.19 ± 3.66	25.17 ± 3.63	25.24 ± 3.73	0.425
WHtR, mean ± SD	0.48 ± 0.06	0.48 ± 0.06	0.49 ± 0.06	0.262
SBP, mean ± SD, mm Hg	120.30 ± 13.57	119.99 ± 13.43	121.04 ± 13.86	0.004
DBP, mean ± SD, mm Hg	79.58 ± 9.32	79.47 ± 9.30	79.84 ± 9.35	0.140
TG, mean ± SD, mmol/L	1.36 ± 0.73	1.35 ± 0.74	1.38 ± 0.73	0.118
LDL‐C, mean ± SD, mmol/L	4.39 ± 1.04	4.37 ± 1.03	4.46 ± 1.05	0.001
HDL‐C, mean ± SD, mmol/L	1.45 ± 0.41	1.45 ± 0.41	1.43 ± 0.41	0.062
Fasting glucose, mean ± SD, mmol/L	5.19 ± 0.47	5.19 ± 0.47	5.19 ± 0.46	0.531
TyG index, mean ± SD	8.50 ± 0.53	8.49 ± 0.53	8.52 ± 0.52	0.042
Family history of diabetes, *N* (%)	697 (10.8)	480 (10.7)	217 (11.2)	0.515
Smoking status, *N* (%)				0.219
Never smoker	3069 (47.7)	2121 (47.1)	948 (49.1)	
Ex‐smoker	2476 (38.5)	1764 (39.2)	712 (36.9)	
Current smoker	886 (13.8)	617 (13.7)	269 (13.9)	
Drinking, *N* (%)				0.433
Non‐current	1173 (18.2)	818 (18.2)	355 (18.4)	
Current moderate	3676 (57.2)	2595 (57.6)	1081 (56.0)	
Current heavy	1582 (24.6)	1089 (24.2)	493 (25.6)	
Fruit/vegetable consumption, *N* (%)				0.142
< Daily	2446 (38.0)	1739 (38.6)	707 (36.7)	
≥ Daily	3985 (62.0)	2763 (61.4)	1222 (63.3)	
Physical activity, *N* (%)				0.033
Inactive	1244 (19.3)	907 (20.1)	337 (17.5)	
Moderate	2309 (35.9)	1613 (35.8)	696 (36.1)	
Active	2878 (44.8)	1982 (44.0)	896 (46.4)	
Socioeconomic status, *N* (%)				0.987
Low	975 (15.2)	681 (15.1)	294 (15.2)	
Intermediate	2936 (45.6)	2058 (45.7)	878 (45.5)	
High	2520 (39.2)	1763 (39.2)	757 (39.2)	
Baseline CVD diagnosis, *N* (%)	593 (9.2)	391 (8.7)	202 (10.5)	0.026
CVD medication, *N* (%)	513 (8.0)	342 (7.6)	171 (8.9)	0.095

*Note: Drinking status*: current moderate (1–14 units/week), current heavy (> 14 units/week); *Socioeconomic status*: based on last recorded civil service employment grade at baseline; low (clerical or support grades), intermediate (professional or executive grades), and high (administrative grades); *Physical activity*: inactive (< 1 h/week of moderate physical activity and < 1 h/week of vigorous physical activity), active (> 2.5 h/week of moderate physical activity or > 1 h/week of vigorous physical activity), or moderate (if not active or inactive); *Baseline CVD diagnosis*: self‐reported physician‐diagnosed CVD, including coronary heart disease, stroke, and other cardiovascular diseases such as heart failure, arrhythmia, and valvular heart disease; CVD medication: use of medications for cardiovascular disease, such as lipid‐lowering medication, antianginal medication, and antihypertensive medication; TyG index = ln(fasting triglycerides [mg/dL] × fasting plasma glucose [mg/dL]/2).

Abbreviations: BMI, body mass index; CVD, cardiovascular disease; DBP, diastolic blood pressure; HDL‐C, high‐density lipoprotein cholesterol; LDL‐C, low‐density lipoprotein cholesterol; SBP, systolic blood pressure; SD, standard deviation; TG, triglycerides; TyG index, triglyceride‐glucose index; WHII, Whitehall II; WHtR, waist‐to‐height ratio.

^a^ *p*‐Values indicate the comparison between WHII Training set and WHII Test set. Continuous variables were compared using the *t*‐test, and categorical variables using the chi‐square test.

#### 3.1.2 Selection of Key Variables

In the Training set, the three methods selected overlapping sets of predictors, including key demographic, anthropometric and metabolic variables. Using LASSO, nine predictors of incident diabetes were selected: ethnicity, WHtR, height, TyG index, fasting glucose, family history of diabetes, smoking, BMI and SES. These variables were selected as they appeared in ≥ 70% of the samples, and the selected set remained unchanged when applying thresholds of 50%, 60% and 80% (Table S7). Using RSF, 13 predictors were selected: age, ethnicity, BMI, height, SBP, DBP, family history of diabetes, SES, WHtR, TG, HDL‐C, fasting glucose and TyG index. Variable importance rankings from the RSF model are shown in Figure S1. By best subset selection, 12 predictors were selected: age, sex, ethnicity, smoking, physical activity, DBP, family history of diabetes, CVD medication, WHtR, fasting glucose, TyG index and HDL‐C. This subset had the lowest AIC. Three candidate Cox models were then developed in the Training set, each using predictors selected by LASSO, RSF, or best subset selection.

#### 3.1.3 Validation of the Selected Variables

The three simplified models showed broadly comparable discrimination to the full model (21 variables) in both the WHII Training and Test sets. For example, in the WHII Test set at 10 years, the *C*‐index was 0.792 (95% CI 0.736–0.844) for the RSF‐selected model, 0.792 (95% CI 0.737–0.843) for the LASSO‐selected model and 0.788 (95% CI 0.731–0.841) for the best subset‐selected model, compared with 0.791 (95% CI 0.734–0.843) for the full model (Table S8). Time‐dependent *C*‐index curves showed consistent patterns across follow‐up time (Figure S2). All three candidate models demonstrated broadly comparable calibration graphically (Figure S3) and quantitatively (Table S9).

### 3.2 Evaluation of Candidate Models in WHII Test Set and ELSA Dataset

#### 3.2.1 Characteristics of Included Participants in External ELSA Dataset

A total of 2190 participants without diabetes at Wave 2 of ELSA were included in the analysis. The mean age was 63.08 years (SD: 7.10), and 1232 (56.3%) were female. There were 255 incident diabetes cases recorded, with a median follow‐up of 14.2 years. Compared with the WHII Training set, ELSA participants were older and included higher proportions of women and White participants, with higher baseline BMI and WHtR (Table S10).

#### 3.2.2 Discrimination

The three models showed similar discrimination in both WHII Test set and ELSA dataset, with RSF and LASSO performing slightly better than best subset selection. For example, in ELSA validation at 10 years, the *C*‐index was 0.822 (95% CI 0.790–0.851) for the RSF model, 0.820 (95% CI 0.786–0.849) for the LASSO model and 0.808 (95% CI 0.773–0.839) for the best subset model (Table 2). Time‐dependent *C*‐index curves showed consistent patterns across follow‐up time (Figure S4).

**TABLE 2:** Time‐dependent C‐index for the candidate models (internal and external validation sets).

	5 years	10 years	15 years
	C‐index (95% CI)	C‐index (95% CI)	C‐index (95% CI)
WHII internal Test set			
LASSO	0.753 (0.647, 0.843)	0.792 (0.737, 0.843)	0.806 (0.772, 0.838)
Random survival forests	0.764 (0.657, 0.853)	0.792 (0.736, 0.844)	0.804 (0.770, 0.836)
Best subset selection	0.747 (0.635, 0.843)	0.788 (0.731, 0.841)	0.802 (0.768, 0.836)
ELSA external dataset			
LASSO	0.878 (0.844, 0.908)	0.820 (0.786, 0.849)	0.784 (0.753, 0.814)
Random survival forests	0.876 (0.843, 0.908)	0.822 (0.790, 0.851)	0.785 (0.756, 0.813)
Best subset selection	0.867 (0.829, 0.902)	0.808 (0.773, 0.839)	0.770 (0.737, 0.802)

*Note: Candidate model for LASSO selection (9 variables)* includes ethnicity, WHtR, height, TyG index, fasting glucose, family history of diabetes, smoking, BMI, and socioeconomic status. *Candidate model for random survival forests selection (13 variables)* includes age, ethnicity, BMI, height, SBP, DBP, family history of diabetes, socioeconomic status, WHtR, TG, HDL‐C, fasting glucose, and TyG index. *Candidate model for best subset selection (12 variables)* includes age, sex, ethnicity, smoking, physical activity, DBP, family history of diabetes, CVD medication, WHtR, fasting glucose, TyG index, and HDL‐C. TyG index = ln(fasting triglycerides [mg/dL] × fasting plasma glucose [mg/dL]/2).

Abbreviations: BMI, body mass index; CI, confidence interval; CVD, cardiovascular disease; DBP, diastolic blood pressure; HDL‐C, high‐density lipoprotein cholesterol; SBP, systolic blood pressure; TG, triglycerides; TyG index, triglyceride‐glucose index; WHII, Whitehall II; WHtR, waist‐to‐height ratio.

#### 3.2.3 Decision Curve Analysis

The three models showed comparable net benefit in both WHII Test set and ELSA dataset (Figure S5). In WHII Test set, net benefit was similar at the prespecified thresholds (25%, 50%, 75%). At 5 and 10 years, net benefit over the “none” strategy was limited at 50% and 75%, with higher values at 25%. In ELSA, net benefit was broadly similar across models at 5 and 10 years. At 15 years, RSF and LASSO exceeded best subset at the prespecified thresholds. For example, at the 25% threshold net benefits were 0.026, 0.023 and 0.021 for RSF, LASSO and best subset selection, respectively.

### 3.3 Transforming Models Into Prediction Tools

The LASSO‐ and RSF‐selected models showed similar performance, with both performing slightly better than the model using best subset selection. These two models were further translated into prediction tools. Linearity assessment identified evidence of non‐linearity for BMI in the LASSO‐selected model and for BMI, TyG and TG in the RSF‐selected model (Table S11). Further evaluation showed comparable predictive performance between the linear and restricted cubic spline specifications (Tables S12 and S13). Therefore, the linear specifications were retained. All VIFs were below 5 in the LASSO‐selected model, whereas VIFs exceeded 5 only for TyG index (15.24) and TG (13.12) in the RSF‐selected model (Table S14).

#### 3.3.1 Risk Score and Risk Group

According to the LASSO‐selected model, the risk score was derived as follows:


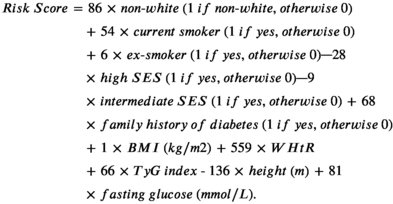


The score ranged from 724.5 to 1449.2 in the Training set and was divided into four risk categories by quartiles: very low (< 985), low (985–1046), medium (1046–1114) and high (≥ 1114). Each cut‐off value was assigned to the group with the higher score range. Validation yielded *C*‐indices of 0.786 (95% CI 0.754–0.817) in WHII Test set and 0.800 (95% CI 0.773–0.827) in ELSA for the risk score, and 0.759 (95% CI 0.730–0.788) and 0.737 (95% CI 0.715–0.759) for the risk group categories. Predicted risks in the Training set and observed diabetes incidence in the WHII Test set and ELSA at 5, 10 and 15 years are shown in Table S15. In all three datasets, diabetes incidence generally increased from the very‐low‐ to the high‐risk group (Figure S6). Separation was clear between the medium‐ and high‐risk groups but relatively limited between the very‐low‐ and low‐risk groups.

According to the RSF‐selected model, the risk score was derived as follows:

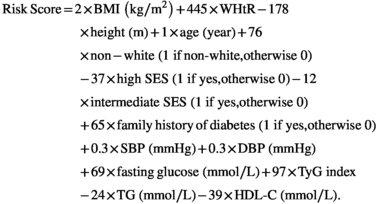




The score ranged from 829.8 to 1563.7 in the Training set and was divided into quartiles: very low (< 1086), low (1086–1152), medium (1152–1220) and high (≥ 1220) risk. Validation showed *C*‐indices of 0.782 (95% CI 0.750–0.814) in WHII Test set and 0.801 (95% CI 0.774–0.827) in ELSA for the risk score, and 0.750 (95% CI 0.721–0.780) and 0.727 (95% CI 0.707–0.748) for the risk groups. Predicted risks in the Training set and observed diabetes incidence in the WHII Test set and ELSA are shown in Table S16. Diabetes incidence generally increased from the very‐low‐ to the high‐risk group in all three datasets (Figure S7). As with the LASSO‐selected model, separation was clear between the medium‐ and high‐risk groups but limited between the very‐low‐ and low‐risk groups. Further details on risk stratification and cumulative diabetes incidence at 5, 10 and 15 years are provided in Table S17.

#### 3.3.2 Nomogram

Figure 2 shows nomograms from Cox models using LASSO‐ and RSF‐selected predictors to estimate 5‐, 10‐ and 15‐year diabetes risk. For each individual, the value of each predictor is located on its corresponding axis and converted to points using the top scale. The points are summed on the “Total Points” axis and converted to 5‐, 10‐ and 15‐year predicted risks using the corresponding bottom scales.

**FIGURE 2:**
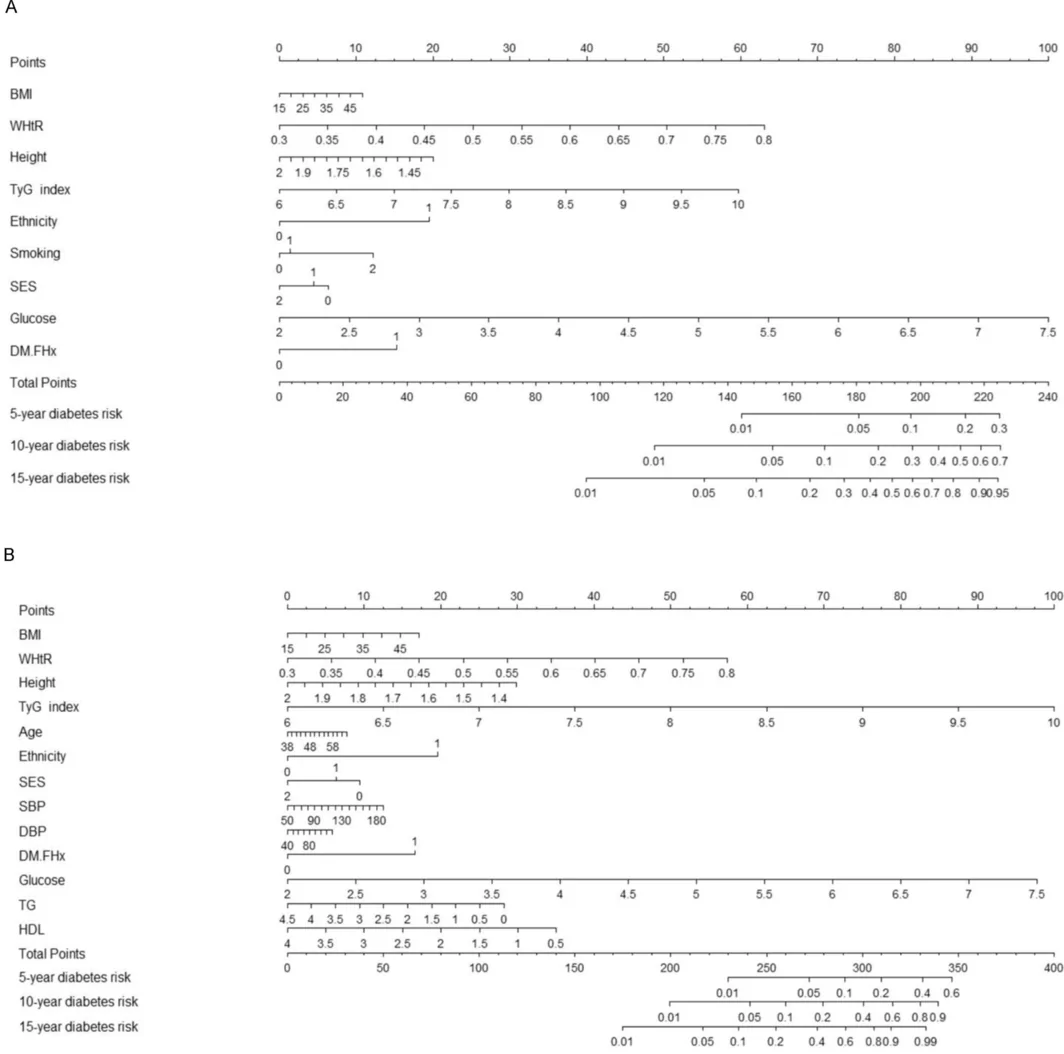
Nomogram for predicting 5‐, 10‐, and 15‐year diabetes risk (A) LASSO‐selected model; (B) RSF‐selected model. BMI, body mass index; DBP, diastolic blood pressure; DM.FHx, family history of diabetes; HDL, high‐density lipoprotein cholesterol; RSF, random survival forests; SBP, systolic blood pressure; SES, socioeconomic status; TG, triglycerides; TyG index, triglyceride‐glucose index; WHtR, waist‐to‐height ratio; Glucose refers to fasting plasma glucose. Ethnicity: White = 0, non‐white = 1; SES: Low = 0, intermediate = 1, high = 2; DM.FHx: no = 0, yes = 1; Smoking: never smoker = 0, ex‐smoker = 1, current smoker = 2. Age: years; BMI: kg/m^2^; Height: m; SBP and DBP: mm Hg; fasting glucose: mmol/L; TG: mmol/L; HDL‐C: mmol/L; TyG index = ln(fasting triglycerides [mg/dL] × fasting plasma glucose [mg/dL]/2).

#### 3.3.3 Additional Analysis Addressing Potential Selection‐Induced Optimism

As an additional analysis, the best subset‐selected model was translated into a prediction tool using the same procedures to derive the risk score, risk groups and nomogram. The corresponding tool and performance results are provided in Tables S18–S21 and Figure S8.

### 3.4 Sensitivity Analysis

In non‐invasive models with BMI as the adiposity index, adding WHtR led to a clear improvement in the *C*‐index (Tables S22 and S23). In models that also include blood measures, adding WHtR yielded only small gains in discrimination. The largest increases in *C*‐index were observed after adding blood measures, regardless of whether the baseline model included BMI, WHtR, or both.

Following multiple imputation of the WHII data, external evaluation in ELSA yielded risk‐score *C*‐indices of 0.798 (95% CI 0.771–0.826) for the LASSO‐selected model and 0.799 (95% CI 0.772–0.826) for the RSF‐selected model. These *C*‐indices were similar to those in the primary complete‐case analysis.

## 4 Discussion

### 4.1 Main Findings

This study developed and validated simple tools to predict 5‐, 10‐ and 15‐year diabetes risk in British middle‐aged adults. Using long‐term prospective data, we applied three variable selection methods, evaluated models using discrimination, calibration, and DCA, and validated risk scores and groups in internal and external datasets. In ELSA, the RSF‐selected model achieved a slightly higher *C*‐index for risk scores, whereas the LASSO‐selected model performed better when scores were grouped. As risk grouping is commonly used in practice to classify individuals into risk categories, the LASSO‐selected model may be more suitable for routine use, while the RSF‐selected model may be useful for more detailed individual risk estimates at 5, 10 and 15 years. Both models included ethnicity, WHtR, height, family history, BMI, SES, TyG index and fasting glucose; LASSO also included smoking, while RSF additionally included age, TG, HDL‐C, SBP and DBP.

In the present study, the three methods selected several common predictors, and the resulting models showed generally comparable performance. This provides some reassurance that the findings were not driven by one selection method. The LASSO and RSF models were retained as the two main tools. This decision considered overall performance rather than a single metric. Overall, the LASSO‐selected tool required fewer predictors and may be easier to implement in primary care. Given the small performance differences, claims of relative superiority should be made cautiously.

### 4.2 Comparison With Existing Studies

Previous studies have developed several diabetes prediction models [10, 11, 14, 36, 37], mostly cross‐sectional. Even among those based on cohort studies, the reporting of the area under the ROC curve (AUC) suggests that predictions were made for a fixed time point [7, 11, 13, 14, 36]. These existing models generally exhibit moderate to good discrimination, with AUCs ranging from approximately 0.70 to 0.85. Since our study used time‐to‐event data, the *C*‐index is more suitable than AUC for assessing discrimination. This limits direct comparison with previous models reporting AUC.

The predictive metrics in this study, including calibration and a *C*‐index of 0.78–0.80, suggested that the present prediction model showed reasonable performance and was generally comparable to studies reporting the same metrics [9, 38]. For instance, Rosella et al. developed a tool (Diabetes Population Risk Tool, DPoRT) to predict the 9‐year risk of diabetes, demonstrating good discriminatory ability (*C* = 0.77–0.80), which is similar to our results [9]. Some important variables, such as fasting blood glucose, were excluded from their tool as they were not collected in the surveys. However, several studies showed that incorporating fasting blood glucose into the model may significantly improve prediction accuracy [8, 12], which supports our findings. In the sensitivity analyses of our study, adding blood‐based variables, including fasting glucose, increased the *C*‐index by about 0.05. Compared with the *C*‐index reported for QDiabetes‐2018 risk prediction algorithm [8], the present tool showed a lower *C*‐index. However, the reported *C*‐index values were derived from different study populations rather than from a direct comparison within the same population and should therefore be interpreted cautiously. The QDiabetes‐2018 model was developed in a substantially larger population (8.87 million), which may have encompassed a broader distribution of individual risk profiles, enhancing its ability to distinguish between individuals with different levels of risk. It also included additional predictors, such as schizophrenia and polycystic ovary syndrome in women, which were not available in Phase 3 WHII. These factors may have contributed to its higher discrimination. The present tool is not intended to replace QDiabetes‐2018, but to offer a complementary, parsimonious approach based on routinely available variables. It combines risk stratification with short‐term and long‐term risk estimates to support assessment across different practice settings and time horizons.

Our prediction model showed a slightly higher *C*‐index in the ELSA dataset than in internal validation. In the present study, the WHII Test set showed a narrower range of risk scores than ELSA, indicating less variation in predicted risk. This difference in the distribution of predicted risk may influence model discrimination, as a wider spread of predicted risk values may allow greater separation between individuals with different outcome probabilities and may partly explain the slightly higher *C*‐index observed in ELSA [39]. The underlying reasons for this finding are not fully clear. Moreover, baseline differences between WHII and ELSA indicate that the model was evaluated in an independent population with a different but relevant participant profile. Although external validation supports potential applicability beyond WHII, these findings should be interpreted with caution. DCA indicated potential clinical utility. In WHII, net benefit was limited at the prespecified thresholds of 50% and 75%, with gains mainly at 25% (the lowest prespecified threshold). This likely reflects low 5‐year diabetes incidence and healthier baseline profiles, meaning few participants exceeded higher thresholds. A similar pattern in ELSA suggests greater clinical utility at lower threshold probabilities.

Sensitivity analyses provide insight into the relative contributions of model components. In non‐invasive models using BMI as the adiposity index, adding WHtR led to some improvement in discrimination, suggesting that WHtR may provide additional information beyond BMI. When blood measures were included, adding WHtR resulted in small gains in discrimination. In contrast, the largest increases in discrimination were observed with the inclusion of blood measures. These findings suggest that blood‐based variables may contribute to predictive performance. The relative contribution of WHtR appears greater when blood measures are not available.

### 4.3 Strengths and Limitations

This study provides 5‐, 10‐ and 15‐year risk estimates, allowing diabetes risk to be assessed across different time periods and supporting prevention planning over time. Presenting risk at different stages provides a broader basis for considering the timing and intensity of preventive strategies than a single risk estimate. This study also stratifies individuals into relative risk groups, helping identify those at higher risk who may warrant closer assessment. Therefore, this study provides quantitative and categorical risk information, which may offer flexibility across different practice settings.

Moreover, this study uses predictors that are routinely available and easily measurable in healthcare settings. This may support its practical use in primary care. Our tool includes measures like WHtR and the TyG index. These were consistently selected in multiple complementary statistical approaches, supporting their inclusion in the final models. WHtR is an alternative to WC and avoids reliance on sex‐specific WC cut‐points. The TyG index is a surrogate marker of insulin resistance derived from fasting triglycerides and glucose [40, 41], both commonly measured in practice. These measures may be practical for large‐scale risk assessment. Their inclusion may provide information on metabolic risk without requiring specialised additional testing. Furthermore, the cumulative incidence of diabetes in this study appears broadly consistent with estimates from the general population in England [42]. Findings from the WHII have also been shown to be broadly generalisable to the general UK population [43]. This may support the relevance of the study findings to the general UK population.

This study has some limitations. Ethnicity in WHII was classified only as white or non‐white, which may obscure differences among non‐white groups. WHII consists of British civil servants and has a limited socioeconomic and ethnic range. Furthermore, employment grade does not capture all aspects of socioeconomic status, such as wealth or social capital. These limitations may reduce generalisability to the broader contemporary UK population. HbA_1c_ was not collected in WHII Phase 3 and was therefore not included in our model, despite being used in some previous models. However, fasting glucose is an established predictor, and the QDiabetes‐2018 model using fasting glucose outperformed the model using HbA_1c_ instead [8]. Future studies with HbA_1c_ data should assess whether adding HbA_1c_ further improves prediction. The inclusion of composite indices alongside their constituent variables may introduce overlapping information and increase the potential for multicollinearity. In the RSF‐selected model, elevated VIFs for TyG index and TG may affect the stability of the risk score across samples, although external validation in ELSA yielded a C‐index of 0.801. Selection‐induced optimism from comparing three candidate models and taking two forward partly based on observed performance cannot be excluded. Reporting the supplementary best subset‐based prediction tool improves transparency but does not remove this concern. Further independent validation would help assess reproducibility. Moreover, the generalisability of the tool to younger adults requires further validation. Both cohorts were England‐based, and applicability outside England requires further evaluation. Predictive performance improved with the inclusion of fasting glucose and lipids; fasting glucose was selected by all three methods and ranked highest by RSF. The tool therefore requires blood‐based measures, limiting applicability where blood testing is not feasible. The quartile‐based tool separated the high‐risk group clearly but distinguished the very‐low‐ and low‐risk groups less clearly. Additionally, in ELSA, diabetes was self‐reported, which may introduce outcome misclassification and influence discrimination estimates in external validation. The number of incident cases was modest, particularly at long follow‐up, which may affect the precision of model evaluation. Sex‐stratified analyses were not performed due to limited sample size and number of events after data splitting. Future studies with larger sample sizes may allow investigation of sex‐specific models. Selection bias from complete‐case analysis cannot be excluded, although C‐indices were similar after multiple imputation. Finally, this study used baseline measurements to predict long‐term risk and did not account for changes in time‐varying factors during follow‐up. Competing risk analysis was not performed; treating death as censoring may have overestimated absolute long‐term diabetes risk, particularly among older individuals.

## 5 Conclusions

Our tool may support diabetes risk assessment and targeted prevention in British middle‐aged adults, with further studies needed to confirm its clinical utility before implementation in practice.

## Funding

The WHII study is supported by the UK Medical Research Council (R024227, S011676); and the Wellcome Trust (221854/Z/20/Z). However, the authors did not receive direct financial support from these sources.

## Disclosure

The authors have nothing to report.

## Conflicts of Interest

The authors declare no conflicts of interest.

## Supporting information


**Table S1:** Cohort descriptions: Whitehall II (WHII) Study and English Longitudinal Study of Ageing (ELSA).
**Table S2:** Missing data for initial candidate predictors in Whitehall II (WHII).
**Table S3:** Definitions of variables requiring additional clarification.
**Table S4:** Supplementary methods for assessing linearity.
**Table S5:** Incident diabetes and follow‐up information in Whitehall II.
**Table S6:** Univariate analyses for testing the association between potential predictors and incident diabetes (Whitehall II).
**Table S7:** LASSO‐Cox modelling results of potential predictors for incident diabetes (Whitehall II).
**Table S8:** Results of time‐dependent *C*‐index (95% CI) for three candidate prediction models (Whitehall II).
**Table S9:** Quantitative calibration metrics for the candidate models in the WHII Training and Test sets.
**Table S10:** Characteristics of included participants in ELSA and comparisons with the WHII Training set.
**Table S11:** Assessment of non‐linearity for continuous predictors in LASSO‐selected and RSF‐selected models in the WHII Training set.
**Table S12:** Sensitivity analysis comparing linear and restricted cubic spline modelling of BMI in the LASSO‐selected model in the WHII Test set.
**Table S13:** Sensitivity analysis comparing linear and restricted cubic spline specifications for BMI, TyG index and TG in the RSF‐selected model in the WHII Test set.
**Table S14:** Variance inflation factors for the LASSO‐ and RSF‐based Cox models in the WHII Training set.
**Table S15:** Predicted and observed diabetes incidence at 5, 10 and 15 years by risk groups according to risk score cut‐off values (LASSO‐selected model).
**Table S16:** Predicted and observed diabetes incidence at 5, 10 and 15 years by risk groups according to risk score cut‐off values (RSF‐selected model).
**Table S17:** Risk score‐based stratification and cumulative diabetes incidence in the WHII Training set, WHII Test set and ELSA dataset.
**Table S18:** Variance inflation factors for the best subset‐selected Cox model in the WHII Training set.
**Table S19:** Assessment of non‐linearity for continuous predictors in the best subset‐selected model in the WHII Training set.
**Table S20:** Comparison of linear and restricted cubic spline specifications for fasting glucose in the best subset‐selected model in the WHII Test set.
**Table S21:** Risk score, risk‐group definitions and discrimination of the prediction tool based on the best subset‐selected model.
**Table S22:** Discrimination performance of LASSO‐selected prediction models: *C*‐index and pairwise comparisons (Whitehall II Training set).
**Table S23:** Discrimination performance of RSF‐selected prediction models: *C*‐index and pairwise comparisons (Whitehall II Training set).
**Figure S1:** Out‐of‐bag (OOB) prediction error plot and random forest variable importance plot (Whitehall II Training set).
**Figure S2:** Time‐dependent concordance index curves for three candidate prediction models in Whitehall II.
**Figure S3:** Calibration plots for 5‐, 10‐ and 15‐year diabetes‐free survival following the simplified model after bootstrapping (Whitehall II).
**Figure S4:** Time‐dependent concordance index curves for simplified models (internal and external validation sets).
**Figure S5:** Decision curve analysis for validation and comparison of the candidate simplified models in WHII Test and ELSA datasets.
**Figure S6:** Kaplan–Meier curves for incident diabetes by risk group in the WHII Training set, WHII Test set and ELSA (LASSO‐selected model).
**Figure S7:** Kaplan–Meier curves for incident diabetes by risk group in the WHII Training set, WHII Test set and ELSA (RSF‐selected model).
**Figure S8:** Nomogram for predicting 5‐, 10‐ and 15‐year diabetes risk (additional analysis for the best subset‐selected model).

## Data Availability

The WHII data and the ELSA data supporting the results of this study are not publicly available but can be accessed upon application to the respective data custodians. Details on data access for the WHII study are available at https://www.ucl.ac.uk/psychiatry/research/mental‐health‐older‐people/whitehall‐ii/data‐sharing. Details for the ELSA study are available at https://www.elsa‐project.ac.uk/accessing‐elsa‐data.
