# Supplementary material for: Improving Short‐ and Long‐Term Type 2 Diabetes Risk Prediction for Middle‐Aged British Adults
Source: Diabetes Obes Metab. 2026 Aug 26;28(11):10574–85. doi: 10.1111/dom.71289 (PMC13646339; doi:10.1111/dom.71289)
Supplement: Supplementary file 1 [file DOM-28-10574-s001.docx]

**Table S1 Cohort descriptions: Whitehall II (WHII) Study and English Longitudinal Study of Ageing (ELSA)**

| **Cohort** | **Descriptions** |
| --- | --- |
| **Whitehall II (WHII) Study** | The WHII study is an ongoing cohort that enrolled 10308 British civil servants (aged 35-55 years; 33.1% women) from 20 London offices in 1985-1988. At enrolment (Phase 1), participants underwent a clinical examination and completed a self-administered questionnaire on demographics, health status, and lifestyle. Subsequent data collection alternated between questionnaire-only phases and phases combining questionnaires with clinical examinations. The WHII received approval from the University College London Research Ethics Committee and adheres to the Declaration of Helsinki. |
| **English Longitudinal Study of Ageing (ELSA)** | ELSA is a prospective cohort study of adults aged 50 years and older living in the UK, initiated in 2002-2003 (Wave 1) with the recruitment of a nationally representative sample of 11391 participants. Follow-up interviews have been conducted every two years to collect socio-demographic, health, and behavioural information. Nurse visits were conducted every four years starting from Wave 2 (i.e., Waves 2, 4, 6, and 8), during which anthropometric measurements were taken and blood samples collected. ELSA adheres to the Declaration of Helsinki; ethical approval was obtained from the National Research Ethics Service and informed consent from all participants. |

Overall, the WHII study is an occupational cohort of British civil servants. ELSA is a nationally representative cohort of adults aged 50 years and older living in England. Both cohorts collect questionnaire data together with clinical and biomarker measurements.

**Table S2 Missing data for initial candidate predictors in Whitehall II (WHII)**

|  | Persons without diabetes in WHII Phase 3 (7488)  Missing, N (%) |
| --- | --- |
| Age | 0 (0) |
| Sex | 0 (0) |
| Ethnicity | 20 (0.3) |
| BMI | 0 (0) |
| WHtR | 0 (0) |
| Height | 0 (0) |
| TyG index | 377 (5.0) |
| Fasting glucose | 377 (5.0) |
| Family history of diabetes | 116 (1.5) |
| Smoking | 543 (7.3) |
| Drinking | 131 (1.7) |
| Socioeconomic status | 129 (1.7) |
| Physical activity | 124 (1.7) |
| Fruit/vegetable consumption | 129 (1.7) |
| TG | 60 (0.8) |
| LDL-C | 195 (2.6) |
| HDL-C | 82 (1.1) |
| SBP | 15 (0.2) |
| DBP | 16 (0.2) |
| Baseline CVD diagnosis | 0 (0) |
| CVD medication | 0 (0) |

Of 7488 WHII participants without diabetes at Phase 3, 1057 (14.1%) had missing data for at least one initial candidate predictor and were excluded from the primary complete-case analysis, leaving 6431 participants.

WHII, Whitehall II; BMI, body mass index; WHtR, waist-to-height ratio; CVD, cardiovascular disease; TG, triglycerides; LDL-C, low-density lipoprotein cholesterol; HDL-C, high-density lipoprotein cholesterol; TyG index, triglyceride-glucose index; SBP, systolic blood pressure; DBP, diastolic blood pressure;

TyG index = ln(fasting triglycerides [mg/dL] × fasting plasma glucose [mg/dL]/2).

**Table S3 Definitions of variables requiring additional clarification**

| **Variable** | **Description** |
| --- | --- |
| **Drinking status** | Current moderate (1-14 units/week), current heavy (> 14 units/week). |
| **Socioeconomic status (SES)** | Based on last recorded civil service employment grade at baseline; low (clerical or support grades), intermediate (professional or executive grades), and high (administrative grades). |
| **Physical activity** | Inactive (< 1 h/week of moderate physical activity and < 1 h/week of vigorous physical activity), active (> 2.5 h/week of moderate physical activity or > 1 h/week of vigorous physical activity), or moderate (if not active or inactive). |
| **Baseline CVD diagnosis** | Self-reported physician-diagnosed CVD, including coronary heart disease, stroke, and other cardiovascular diseases such as heart failure, arrhythmia, and valvular heart disease. |
| **CVD medication** | Use of medications for cardiovascular disease, such as lipid-lowering medication, antianginal medication, and antihypertensive medication. |

CVD, cardiovascular disease.

**Table S4 Supplementary methods for assessing linearity**

| Linearity assessment of continuous predictors in the candidate models | The linearity of continuous predictors was assessed in the WHII Training set using restricted cubic splines with four knots, with non-linearity evaluated using Wald tests. For predictors showing evidence of non-linearity, sensitivity models incorporating restricted cubic spline (RCS) terms were compared with the corresponding models containing linear terms using the Akaike information criterion (AIC). Time-dependent C-index and observed-to-expected (O/E) ratios at 5, 10, and 15 years were compared in the WHII Test set, with 95% confidence intervals obtained from 1000 bootstrap samples. |
| --- | --- |

WHII, Whitehall II.

**Table S5 Incident diabetes and follow-up information in Whitehall II**

| **Characteristic** | **All, N (%)**  **N = 6431 (100)** | **WHII Training set, N (%)**  **N = 4502 (70)** | **WHII Test set, N (%)**  **N = 1929 (30)** | **P value** ^†^ |
| --- | --- | --- | --- | --- |
| **Number of diabetes** | 782 (12.2) | 547 (12.2) | 235 (12.2) | 0.971 |
| **Follow-up time** |  |  |  |  |
| **Mean ± SD, yrs** | 14.34 ± 3.90 | 14.34 ± 3.88 | 14.32 ± 3.96 | 0.879 |
| **Median (IQR), yrs** | 16.1 (14.6-16.5) | 16.1 (14.5-16.5) | 16.1 (14.5-16.5) |  |

^†^ P-value for the comparison between Training set and Test set. Continuous variables were compared using the t-test, and categorical variables using the chi-square test.

SD, standard deviation; IQR, interquartile range.

**Table S6 Univariate analyses for testing the association between potential predictors and incident diabetes (Whitehall II)**

| **Characteristic** | **HR (95% CI)** | **P value** |
| --- | --- | --- |
| **Age, yrs** | 1.04 (1.03, 1.05) | < 0.001 |
| **Sex** | 1.17 (1.01, 1.36) | 0.038 |
| **Ethnicity (Non-white)** | 3.15 (2.63, 3.78) | < 0.001 |
| **Height, cm** | 0.98 (0.97, 0.98) | < 0.001 |
| **BMI, kg/m^2^** | 1.14 (1.13, 1.16) | < 0.001 |
| **WHtR, Per 0.01 increase** | 1.11 (1.10, 1.12) | < 0.001 |
| **SBP, mm Hg** | 1.02 (1.02, 1.03) | < 0.001 |
| **DBP, mm Hg** | 1.03 (1.03, 1.04) | < 0.001 |
| **TG, mmol/L** | 1.80 (1.67, 1.93) | < 0.001 |
| **LDL-C, mmol/L** | 1.20 (1.12, 1.28) | < 0.001 |
| **HDL-C, mmol/L** | 0.37 (0.30, 0.45) | < 0.001 |
| **Fasting glucose, mmol/L** | 3.37 (2.92, 3.89) | < 0.001 |
| **TyG index** | 3.39 (2.97, 3.87) | < 0.001 |
| **Family history of diabetes** | 2.48 (2.09, 2.94) | < 0.001 |
| **Smoking Status** |  |  |
| Never smoker | Reference |  |
| Ex-smoker | 1.11 (0.95, 1.29) | 0.202 |
| Current smoker | 1.58 (1.30, 1.93) | < 0.001 |
| **Drinking** |  |  |
| Non-current | Reference |  |
| Current moderate | 0.67 (0.56, 0.80) | < 0.001 |
| Current heavy | 0.71 (0.58, 0.87) | 0.001 |
| **Fruit/vegetable consumption** | 0.74 (0.64, 0.85) | < 0.001 |
| **Physical activity** |  |  |
| Inactive | Reference |  |
| Moderate | 0.82 (0.68, 0.99) | 0.040 |
| Active | 0.70 (0.58, 0.84) | < 0.001 |
| **Socioeconomic status** |  |  |
| Low | Reference |  |
| Intermediate | 0.66 (0.55, 0.79) | < 0.001 |
| High | 0.45 (0.37, 0.55) | < 0.001 |
| **Baseline CVD diagnosis** | 1.39 (1.12, 1.73) | 0.003 |
| **CVD medication** | 2.13 (1.74, 2.60) | < 0.001 |

HR, hazard ratio; CI, confidence interval; BMI, body mass index; WHtR, waist-to-height ratio; CVD, cardiovascular disease; TG, triglycerides; LDL-C, low-density lipoprotein cholesterol; HDL-C, high-density lipoprotein cholesterol; TyG index, triglyceride-glucose index; SBP, systolic blood pressure; DBP, diastolic blood pressure.

TyG index = ln(fasting triglycerides [mg/dL] × fasting plasma glucose [mg/dL]/2).

**Table S7 LASSO-Cox modelling results of potential predictors for incident diabetes (Whitehall II)**

|  | Frequency (%) selected in the LASSO model |
| --- | --- |
| **Selected variables** |  |
| Ethnicity | 100 |
| WHtR | 100 |
| Height | 100 |
| TyG index | 100 |
| Fasting glucose | 100 |
| Family history of diabetes | 100 |
| Smoking | 95.8 |
| BMI | 92.9 |
| Socioeconomic status | 86.9 |
| **Unselected variables** |  |
| Sex | 48.3 |
| CVD medication | 48.3 |
| Physical activity | 31.8 |
| HDL-C | 18.3 |
| Age | 7.1 |
| SBP | 2.0 |
| Drinking | 0 |
| Fruit/vegetable consumption | 0 |
| Baseline CVD diagnosis | 0 |
| DBP | 0 |
| TG | 0 |
| LDL-C | 0 |

BMI, body mass index; WHtR, waist-to-height ratio; CVD, cardiovascular disease; TG, triglycerides; LDL-C, low-density lipoprotein cholesterol; HDL-C, high-density lipoprotein cholesterol; TyG index, triglyceride-glucose index; SBP, systolic blood pressure; DBP, diastolic blood pressure;

TyG index = ln(fasting triglycerides [mg/dL] × fasting plasma glucose [mg/dL]/2).

**Table S8** **Results of time-dependent C-index (95% CI) for three candidate prediction models (Whitehall II)**

|  | 5 years | 10 years | 15 years |
| --- | --- | --- | --- |
| **Training set (Whitehall II)** |  |  |  |
| Full model | 0.764 (0.667, 0.850) | 0.782 (0.730, 0.836) | 0.771 (0.739, 0.803) |
| Candidate model (LASSO) | 0.773 (0.677, 0.859) | 0.783 (0.734, 0.837) | 0.771 (0.737, 0.803) |
| Candidate model (random survival forests) | 0.776 (0.689, 0.861) | 0.791 (0.743, 0.842) | 0.774 (0.743, 0.805) |
| Candidate model (best subset selection) | 0.765 (0.670, 0.854) | 0.782 (0.732, 0.834) | 0.773 (0.742, 0.805) |
| **Test set (Whitehall II)** |  |  |  |
| Full model | 0.754 (0.640, 0.847) | 0.791 (0.734, 0.843) | 0.803 (0.769, 0.836) |
| Candidate model (LASSO) | 0.753 (0.647, 0.843) | 0.792 (0.737, 0.843) | 0.806 (0.772, 0.838) |
| Candidate model (random survival forests) | 0.764 (0.657, 0.853) | 0.792 (0.736, 0.844) | 0.804 (0.770, 0.836) |
| Candidate model (best subset selection) | 0.747 (0.635, 0.843) | 0.788 (0.731, 0.841) | 0.802 (0.768, 0.836) |

Full model includes age, sex, ethnicity, height, BMI, WHtR, SBP, DBP, TG, LDL-C, HDL-C, TyG index, fasting glucose, family history of diabetes, smoking, drinking, fruit/vegetable consumption, physical activity, socioeconomic status, baseline CVD diagnosis, and CVD medication (21 variables).

Candidate model (LASSO) includes ethnicity, WHtR, height, TyG index, fasting glucose, family history of diabetes, smoking, BMI, and socioeconomic status (9 variables).

Candidate model (random survival forests) includes age, ethnicity, BMI, height, SBP, DBP, family history of diabetes, socioeconomic status, WHtR, TG, HDL-C, fasting glucose, and TyG index (13 variables).

Candidate model (best subset selection) includes age, sex, ethnicity, smoking, physical activity, DBP, family history of diabetes, CVD medication, WHtR, fasting glucose, TyG index, and HDL-C (12 variables).

CI, confidence interval; BMI, body mass index; WHtR, waist-to-height ratio; TG, triglycerides; LDL-C, low-density lipoprotein cholesterol; HDL-C, high-density lipoprotein cholesterol; TyG index, triglyceride-glucose index; SBP, systolic blood pressure; DBP, diastolic blood pressure; CVD, cardiovascular disease;

TyG index = ln(fasting triglycerides [mg/dL] × fasting plasma glucose [mg/dL]/2).

**Table S9 Quantitative calibration metrics for the candidate models in the WHII Training and Test sets**

| **Model** | **Calibration slope**  **(95% CI)** | **O/E ratio at 5 years**  **(95% CI)** | **O/E ratio at 10 years**  **(95% CI)** | **O/E ratio at 15 years**  **(95% CI)** |
| --- | --- | --- | --- | --- |
| **WHII Training set** | | | | |
| LASSO-selected | 1.000 (0.919, 1.080) | 0.998 (0.754, 1.244) | 0.991 (0.856, 1.128) | 0.980 (0.894, 1.071) |
| RSF-selected | 1.000 (0.919, 1.081) | 0.998 (0.746, 1.253) | 0.991 (0.859, 1.138) | 0.980 (0.894, 1.069) |
| Best subset-selected | 1.000 (0.921, 1.079) | 0.999 (0.765, 1.254) | 0.990 (0.852, 1.130) | 0.975 (0.890, 1.061) |
| **WHII Test set** | | | | |
| LASSO-selected | 0.968 (0.857, 1.079) | 0.866 (0.537, 1.218) | 1.064 (0.856, 1.291) | 0.946 (0.815, 1.072) |
| RSF-selected | 0.986 (0.872, 1.100) | 0.857 (0.530, 1.248) | 1.055 (0.852, 1.277) | 0.940 (0.810, 1.069) |
| Best subset-selected | 0.896 (0.791, 1.002) | 0.852 (0.530, 1.194) | 1.050 (0.849, 1.275) | 0.935 (0.825, 1.065) |

Calibration-in-the-large was assessed using the observed-to-expected (O/E) ratio at 5, 10, and 15 years. An O/E ratio of 1 and a calibration slope of 1 indicate ideal calibration. O/E ratios below 1 indicate overall overestimation of risk, whereas values above 1 indicate overall underestimation. Training-set estimates represent apparent calibration. The 95% confidence intervals for O/E ratios were obtained from 1000 bootstrap samples.

WHII, Whitehall II; CI, confidence interval; O/E, observed-to-expected; RSF, random survival forests.

**Table S10 Characteristics of included participants in ELSA and comparisons with the WHII Training set**

**Panel A:** Characteristics of included participants in ELSA

| **Characteristics** | **ELSA, N (%)**  **N = 2190 (100)** |
| --- | --- |
| **Age, mean ± SD, yrs** | 63.08 ± 7.10 |
| Age range | (52, 79) |
| **Sex (Female), N (%)** | 1232 (56.3) |
| **Ethnicity (non-white), N (%)** | 33 (1.5) |
| **BMI at baseline (Wave 2 of ELSA), mean ± SD, kg/m^2^** | 27.59 ± 4.61 |
| **WHtR at baseline (Wave 2 of ELSA), mean ± SD** | 0.57 ± 0.07 |
| **Height (Wave 2 of ELSA), mean ± SD, m** | 1.66 ± 0.09 |
| **Follow-up time, median (IQR), yrs** | 14.2 (11.7, 17.4) |
| **Number of events at the end of follow-up** | 255 (11.6) |
| **SBP, mean ± SD, mm Hg** | 133.36 ± 17.97 |
| **DBP, mean ± SD, mm Hg** | 76.55 ± 10.31 |
| **TG, mean ± SD, mmol/L** | 1.57 ± 0.88 |
| **HDL-C, mean ± SD, mmol/L** | 1.58 ± 0.38 |
| **Fasting glucose, mean ± SD, mmol/L** | 4.96 ± 0.70 |
| **TyG index, mean ± SD** | 8.61 ± 0.51 |
| **Family history of diabetes, N (%)** | 305 (13.9) |
| **Smoking Status, N (%)** |  |
| Never smoker | 887 (40.5) |
| Ex-smoker | 1066 (48.7) |
| Current smoker | 237 (10.8) |
| **Physical activity, N (%)** |  |
| Inactive | 401 (18.3) |
| Moderate | 1231 (56.2) |
| Active | 558 (25.5) |
| **Socioeconomic status, N (%)** |  |
| Low | 840 (38.4) |
| Intermediate | 579 (26.4) |
| High | 771 (35.2) |
| **CVD medication, N (%)** | 293 (13.4) |
| **Risk score range (LASSO-selected variables)** | 781.7-1995.3 |
| **Risk group, N (%)** |  |
| Very low risk (< 985) | 269 (12.3) |
| Low risk (985-1046) | 453 (20.7) |
| Medium risk (1046-1114) | 618 (28.2) |
| High risk (≥ 1114) | 850 (38.8) |
| **Risk score range (RSF-selected variables)** | 885.0-2005.7 |
| **Risk group, N (%)** |  |
| Very low risk (< 1086) | 221 (10.1) |
| Low risk (1086-1152) | 424 (19.4) |
| Medium risk (1152-1220) | 579 (26.4) |
| High risk (≥ 1220) | 966 (44.1) |

**Panel B:** Comparison of baseline characteristics between the WHII Training set and ELSA external validation cohort

| **Characteristics** | **WHII Training set, N (%)**  **N = 4502** | **ELSA, N (%)**  **N = 2190** | **P value** ^†^ |
| --- | --- | --- | --- |
| **Age, mean ± SD, yrs** | 49.96 ± 6.00 | 63.08 ± 7.10 | < 0.001 |
| **Sex (Female), N (%)** | 1397 (31.0) | 1232 (56.3) | < 0.001 |
| **Ethnicity (White), N (%)** | 4143 (92.0) | 2157 (98.5) | < 0.001 |
| **BMI, mean ± SD, kg/m^2^** | 25.17 ± 3.63 | 27.59 ± 4.61 | < 0.001 |
| **WHtR, mean ± SD** | 0.48 ± 0.06 | 0.57 ± 0.07 | < 0.001 |
| **Height, mean ± SD, m** | 1.72 ± 0.09 | 1.66 ± 0.09 | < 0.001 |
| **SBP, mean ± SD, mm Hg** | 119.99 ± 13.43 | 133.36 ± 17.97 | < 0.001 |
| **DBP, mean ± SD, mm Hg** | 79.47 ± 9.30 | 76.55 ± 10.31 | < 0.001 |
| **TG, mean ± SD, mmol/L** | 1.35 ± 0.74 | 1.57 ± 0.88 | < 0.001 |
| **HDL-C, mean ± SD, mmol/L** | 1.45 ± 0.41 | 1.58 ± 0.38 | < 0.001 |
| **Fasting glucose, mean ± SD, mmol/L** | 5.19 ± 0.47 | 4.96 ± 0.70 | < 0.001 |
| **TyG index, mean ± SD** | 8.49 ± 0.53 | 8.61 ± 0.51 | < 0.001 |
| **Family history of diabetes, N (%)** | 480 (10.7) | 305 (13.9) | < 0.001 |
| **Smoking Status, N (%)** |  |  | < 0.001 |
| Never smoker | 2121 (47.1) | 887 (40.5) |  |
| Ex-smoker | 1764 (39.2) | 1066 (48.7) |  |
| Current smoker | 617 (13.7) | 237 (10.8) |  |
| **Physical activity, N (%)** |  |  | < 0.001 |
| Inactive | 907 (20.1) | 401 (18.3) |  |
| Moderate | 1613 (35.8) | 1231 (56.2) |  |
| Active | 1982 (44.0) | 558 (25.5) |  |
| **Socioeconomic status, N (%)** |  |  | < 0.001 |
| Low | 681 (15.1) | 840 (38.4) |  |
| Intermediate | 2058 (45.7) | 579 (26.4) |  |
| High | 1763 (39.2) | 771 (35.2) |  |
| **CVD medication, N (%)** | 342 (7.6) | 293 (13.4) | < 0.001 |

^†^ P values indicate the comparison between WHII Training set and ELSA. Continuous variables were compared using the t-test, and categorical variables using the chi-square test.

ELSA, English Longitudinal Study of Ageing; WHII, Whitehall II; SD, standard deviation; BMI, body mass index; WHtR, waist-to-height ratio; RSF, random survival forests; CVD, cardiovascular disease; TG, triglycerides; HDL-C, high-density lipoprotein cholesterol; TyG index, triglyceride-glucose index; SBP, systolic blood pressure; DBP, diastolic blood pressure;

Each cut-off value was assigned to the group with the higher score range;

TyG index = ln(fasting triglycerides [mg/dL] × fasting plasma glucose [mg/dL]/2).

**Table S11 Assessment of non-linearity for continuous predictors in LASSO-selected and RSF-selected models in the WHII Training set**

**Panel A:** LASSO-selected model

| **Predictor** | **P value for non-linearity** |
| --- | --- |
| **Fasting glucose** | 0.104 |
| **TyG index** | 0.167 |
| **Height** | 0.227 |
| **WHtR** | 0.383 |
| **BMI** | 0.015 |

**Panel B:** RSF-selected model

| **Predictor** | **P value for non-linearity** |
| --- | --- |
| **Age** | 0.195 |
| **Fasting glucose** | 0.098 |
| **TyG index** | 0.009 |
| **SBP** | 0.469 |
| **DBP** | 0.187 |
| **HDL-C** | 0.621 |
| **TG** | 0.042 |
| **Height** | 0.252 |
| **WHtR** | 0.145 |
| **BMI** | 0.004 |

WHII, Whitehall II; BMI, body mass index; WHtR, waist-to-height ratio; TG, triglycerides; HDL-C, high-density lipoprotein cholesterol; TyG index, triglyceride-glucose index; SBP, systolic blood pressure; DBP, diastolic blood pressure.

TyG index = ln(fasting triglycerides [mg/dL] × fasting plasma glucose [mg/dL]/2).

**Table S12 Sensitivity analysis comparing linear and restricted cubic spline modelling of BMI in the LASSO-selected model** **in the WHII Test set**

| **Time** | **Linear BMI C-index**  **(95% CI)** | **RCS BMI C-index**  **(95% CI)** | **C-index difference**  **(95% CI)** | **Linear BMI O/E ratio**  **(95% CI)** | **RCS BMI O/E ratio**  **(95% CI)** |
| --- | --- | --- | --- | --- | --- |
| **5 years** | 0.753 (0.649, 0.844) | 0.754 (0.649, 0.846) | 0.001 (-0.006, 0.008) | 0.866 (0.565, 1.229) | 0.863 (0.562, 1.224) |
| **10 years** | 0.792 (0.739, 0.843) | 0.793 (0.739, 0.843) | 0.001 (-0.006, 0.011) | 1.064 (0.868, 1.274) | 1.060 (0.862, 1.267) |
| **15 years** | 0.806 (0.774, 0.838) | 0.806 (0.776, 0.838) | 0.000 (-0.004, 0.006) | 0.946 (0.825, 1.073) | 0.943 (0.822, 1.069) |

BMI was modelled as either a linear term or using restricted cubic splines with four knots.

C-index differences were calculated as RCS minus the linear specification.

WHII, Whitehall II; BMI, body mass index; CI, confidence interval; O/E, observed-to-expected; RCS, restricted cubic spline.

The 95% confidence intervals were obtained using 1000 bootstrap samples.

In the WHII Training set, evidence of non-linearity was observed for BMI in the LASSO-selected model (P for non-linearity = 0.015). The AIC was 8291.424 when BMI was modelled as a linear term and 8287.788 when BMI was modelled using restricted cubic splines. In the WHII Test set, absolute differences in C-index between the linear and RCS specifications for BMI were no greater than 0.001 at 5, 10, or 15 years, and the 95% confidence intervals for these differences all included zero. O/E ratios were also nearly identical between the two specifications. BMI was therefore retained as a linear term to maintain model parsimony and facilitate implementation.

**Table S13 Sensitivity analysis comparing linear and restricted cubic spline specifications for BMI, TyG index, and TG in the RSF-selected model** **in the WHII Test set**

| **Time** | **Linear C-index**  **(95% CI)** | **RCS C-index**  **(95% CI)** | **C-index difference (95% CI)** | **Linear O/E ratio (95% CI)** | **RCS O/E ratio (95% CI)** |
| --- | --- | --- | --- | --- | --- |
| **5 years** | 0.764 (0.661, 0.854) | 0.756 (0.647, 0.849) | -0.008 (-0.021, 0.004) | 0.857 (0.557, 1.217) | 0.856 (0.553, 1.216) |
| **10 years** | 0.792 (0.737, 0.845) | 0.794 (0.742, 0.843) | 0.002 (-0.009, 0.016) | 1.055 (0.864, 1.260) | 1.053 (0.861, 1.257) |
| **15 years** | 0.804 (0.773, 0.837) | 0.803 (0.772, 0.834) | -0.001 (-0.008, 0.007) | 0.940 (0.824, 1.065) | 0.940 (0.824, 1.065) |

BMI, TyG index, and TG were modelled either as linear terms or simultaneously using restricted cubic splines with four knots. C-index differences were calculated as RCS minus the linear specification.

BMI, body mass index; WHII, Whitehall II; CI, confidence interval; O/E, observed-to-expected; RCS, restricted cubic spline; TG, triglycerides; TyG, triglyceride-glucose index.

The 95% confidence intervals were obtained using 1000 bootstrap samples.

Following the linearity assessment in the WHII Training set, BMI, TyG index, and TG were simultaneously modelled using restricted cubic splines in a sensitivity analysis of the RSF-selected model. The AIC was 8302.059 with linear terms and 8295.769 with RCS terms. In the WHII Test set, compared with the linear specification, the RCS specification produced C-indices that were 0.008 lower at 5 years, 0.002 higher at 10 years, and 0.001 lower at 15 years. All 95% confidence intervals for these differences included zero, and O/E ratios were nearly identical between the specifications. The linear terms were therefore retained to maintain model parsimony and facilitate implementation.

**Table S14 Variance inflation factors for the LASSO- and RSF-based Cox models in the WHII Training set**

**Panel A:** LASSO-selected model

| **Predictor** | **VIF** |
| --- | --- |
| **Ethnicity** | 1.32 |
| **WHtR** | 4.73 |
| **Height** | 1.39 |
| **TyG index** | 1.31 |
| **Fasting glucose** | 1.15 |
| **Family history of diabetes** | 1.06 |
| **Smoking** |  |
| Ex-smoker | 1.22 |
| Current smoker | 1.25 |
| **BMI** | 4.53 |
| **SES** |  |
| Intermediate | 2.10 |
| High | 2.48 |

**Panel B:** RSF-selected model

| **Predictor** | **VIF** |
| --- | --- |
| **Ethnicity** | 1.30 |
| **WHtR** | 4.85 |
| **Height** | 1.57 |
| **TyG index** | 15.24 |
| **Fasting glucose** | 1.68 |
| **Family history of diabetes** | 1.05 |
| **BMI** | 4.54 |
| **SES** |  |
| Intermediate | 2.10 |
| High | 2.44 |
| **Age** | 1.14 |
| **SBP** | 2.30 |
| **DBP** | 2.34 |
| **TG** | 13.12 |
| **HDL-C** | 1.47 |

VIF, variance inflation factor; SES, socioeconomic status; BMI, body mass index; WHtR, waist-to-height ratio; TG, triglycerides; HDL-C, high-density lipoprotein cholesterol; TyG index, triglyceride-glucose index; SBP, systolic blood pressure; DBP, diastolic blood pressure.

TyG index = ln(fasting triglycerides [mg/dL] × fasting plasma glucose [mg/dL]/2).

**Table S15 Predicted and observed diabetes incidence at 5, 10, and 15 years by risk groups according to risk score cut-off values (LASSO-selected model)**

**Panel A:** Predicted incidence in the WHII Training set and observed incidence in the WHII Test set

| Risk category | Score interval | Predicted incidence of diabetes (95% CI) in the WHII Training set ^†^ | | | | Observed incidence of diabetes (95% CI) in the WHII Test set | | | |
| --- | --- | --- | --- | --- | --- | --- | --- | --- | --- |
|  |  | n | 5 years | 10 years | 15 years | n | 5 years | 10 years | 15 years |
| Very low | < 985 | 1137 | 0 | 1% | 2% (1.9-2.1%) | 466 | 0 | 1% (0.3-2.4%) | 2% (0.6-3.0%) |
| Low | 985-1046 | 1127 | 1% | 2% | 5% (4.9-5.1%) | 482 | 0 | 2% (0.4-2.6%) | 2% (0.9-3.6%) |
| Medium | 1046-1114 | 1109 | 1% | 3% (2.9-3.1%) | 9% (8.9-9.1%) | 487 | 1% (0.3-2.3%) | 2% (1.0-3.7%) | 7% (5.0-10.0%) |
| High | ≥ 1114 | 1129 | 3% (2.8-3.2%) | 10% (9.5-10.5%) | 23% (22.2-23.8%) | 494 | 3% (1.4- 4.4%) | 13% (10.3-16.6%) | 27% (23.0-31.4%) |

**Panel B:** Predicted incidence in the WHII Training set and observed incidence in ELSA

| Risk category | Score interval | Predicted incidence of diabetes (95% CI) in the WHII Training set ^†^ | | | | Observed incidence of diabetes (95% CI) in the ELSA set | | | |
| --- | --- | --- | --- | --- | --- | --- | --- | --- | --- |
|  |  | n | 5 years | 10 years | 15 years | n | 5 years | 10 years | 15 years |
| Very low | < 985 | 1137 | 0 | 1% | 2% (1.9-2.1%) | 269 | 0 | 0 | 1% (0.0-2.0%) |
| Low | 985-1046 | 1127 | 1% | 2% | 5% (4.9-5.1%) | 453 | 0 | 1% (0.0-1.8%) | 4% (1.5-5.7%) |
| Medium | 1046-1114 | 1109 | 1% | 3% (2.9-3.1%) | 9% (8.9-9.1%) | 618 | 1% (0.4-2.2%) | 4% (2.3-5.4%) | 8% (5.5-10.4%) |
| High | ≥ 1114 | 1129 | 3% (2.8-3.2%) | 10% (9.5-10.5%) | 23% (22.2-23.8%) | 850 | 10% (7.8-11.7%) | 16% (13.4-18.4%) | 24% (20.5-26.9%) |

^†^ Predicted incidence from the simplified Cox model.

Each cut-off value was assigned to the group with the higher score range.

**Table S16** **Predicted and observed diabetes incidence at 5, 10, and 15 years by risk groups according to risk score cut-off values (RSF-selected model)**

**Panel A:** Predicted incidence in the WHII Training set and observed incidence in the WHII Test set

| Risk category | Score interval | Predicted incidence of diabetes (95% CI) in the WHII Training set ^†^ | | | | Observed incidence of diabetes (95% CI) in the WHII Test set | | | |
| --- | --- | --- | --- | --- | --- | --- | --- | --- | --- |
|  |  | n | 5 years | 10 years | 15 years | n | 5 years | 10 years | 15 years |
| Very low | < 1086 | 1128 | 0 | 1% | 2% (1.9-2.1%) | 437 | 0 | 2% (0.4-2.8%) | 2% (0.7-3.5%) |
| Low | 1086-1152 | 1123 | 1% | 2% | 5% (4.9-5.1%) | 507 | 0 | 1% (0.1-1.9%) | 1% (0.4-2.5%) |
| Medium | 1152-1220 | 1119 | 1% | 3% (2.9-3.1%) | 9% (8.9-9.1%) | 489 | 1% (0.1-2.0%) | 3% (1.3-4.3%) | 9% (6.4-12.0%) |
| High | ≥ 1220 | 1132 | 3% (2.8-3.2%) | 10% (9.5-10.5%) | 23% (22.2-23.8%) | 496 | 3% (1.6- 4.7%) | 13% (10.0-16.2%) | 26% (21.6-29.9%) |

**Panel B:** Predicted incidence in the WHII Training set and observed incidence in ELSA

| Risk category | Score interval | Predicted incidence of diabetes (95% CI) in the WHII Training set ^†^ | | | | Observed incidence of diabetes (95% CI) in the ELSA set | | | |
| --- | --- | --- | --- | --- | --- | --- | --- | --- | --- |
|  |  | n | 5 years | 10 years | 15 years | n | 5 years | 10 years | 15 years |
| Very low | < 1086 | 1128 | 0 | 1% | 2% (1.9-2.1%) | 221 | 0 | 0 | 0 |
| Low | 1086-1152 | 1123 | 1% | 2% | 5% (4.9-5.1%) | 424 | 0 | 0 | 2% (0.5-3.7%) |
| Medium | 1152-1220 | 1119 | 1% | 3% (2.9-3.1%) | 9% (8.9-9.1%) | 579 | 1% (0.4-2.3%) | 4% (2.0-5.1%) | 7% (4.9-9.8%) |
| High | ≥ 1220 | 1132 | 3% (2.8-3.2%) | 10% (9.5-10.5%) | 23% (22.2-23.8%) | 966 | 9% (6.8-10.3%) | 15% (12.4-16.9%) | 23% (19.5-25.4%) |

^†^ Predicted incidence from the simplified Cox model.

Each cut-off value was assigned to the group with the higher score range.

**Table S17 Risk score-based stratification and cumulative diabetes incidence in the WHII Training set, WHII Test set, and ELSA dataset**

**Panel A:** Distribution of participants and incident diabetes cases by risk group at the end of follow-up

|  | **WHII Training set, N (Events)**  **N = 4502 (547)** | **WHII Test set, N (Events)**  **N = 1929 (235)** | **ELSA dataset, N (Events)**  **N = 2190 (255)** |
| --- | --- | --- | --- |
| **Risk score range (LASSO-selected variables)** | 724.5-1449.2 | 774.5-1479.7 | 781.7-1995.3 |
| **Risk group, N (Events)** |  |  |  |
| Very low risk (< 985) | 1137 (42) | 466 (13) | 269 (2) |
| Low risk (985-1046) | 1127 (64) | 482 (19) | 453 (14) |
| Medium risk (1046-1114) | 1109 (108) | 487 (58) | 618 (45) |
| High risk (≥ 1114) | 1129 (333) | 494 (145) | 850 (194) |
| **Risk score range (RSF-selected variables)** | 829.8-1563.7 | 809.8-1582.2 | 885.0-2005.7 |
| **Risk group, N (Events)** |  |  |  |
| Very low risk (< 1086) | 1128 (45) | 437 (14) | 221 (1) |
| Low risk (1086-1152) | 1123 (59) | 507 (17) | 424 (9) |
| Medium risk (1152-1220) | 1119 (107) | 489 (63) | 579 (39) |
| High risk (≥ 1220) | 1132 (336) | 496 (141) | 966 (206) |

**Panel B:** Cumulative diabetes incidence at 5, 10, and 15 years by risk group

|  | **5-year incidence (95% CI)** | **10-year incidence (95% CI)** | **15-year incidence (95% CI)** |
| --- | --- | --- | --- |
| **WHII Training set** |  |  |  |
| **LASSO-selected model** |  |  |  |
| Very low risk (< 985) | 0.4% (0.1-0.8%) | 1.2% (0.5-1.8%) | 2.5% (1.6-3.5%) |
| Low risk (985-1046) | 0.3% (0.0-0.6%) | 1.0% (0.4-1.6%) | 3.5% (2.3-4.6%) |
| Medium risk (1046-1114) | 1.0% (0.4-1.6%) | 2.6% (1.6-3.5%) | 7.1% (5.5-8.7%) |
| High risk (≥ 1114) | 3.4% (2.4-4.5%) | 11.6% (9.7-13.5%) | 25.4% (22.7-28.1%) |
| **RSF-selected model** |  |  |  |
| Very low risk (< 1086) | 0.4% (0.0-0.7%) | 1.1% (0.5-1.7%) | 2.6% (1.6-3.5%) |
| Low risk (1086-1152) | 0.4% (0.0-0.7%) | 1.1% (0.5-1.8%) | 3.3% (2.2-4.4%) |
| Medium risk (1152-1220) | 1.0% (0.4-1.6%) | 2.2% (1.3-3.0%) | 6.6% (5.1-8.2%) |
| High risk (≥ 1220) | 3.4% (2.4-4.5%) | 11.9% (10.0-13.9%) | 25.9% (23.1-28.6%) |
| **WHII Test set** |  |  |  |
| **LASSO-selected model** |  |  |  |
| Very low risk (< 985) | 0.2% (0.0-0.6%) | 1.3% (0.3-2.3%) | 1.8% (0.5-3.0%) |
| Low risk (985-1046) | 0.4% (0.0-1.0%) | 1.5% (0.4-2.6%) | 2.2% (0.9-3.6%) |
| Medium risk (1046-1114) | 1.3% (0.3-2.3%) | 2.4% (1.0-3.7%) | 7.5% (4.9-10.0%) |
| High risk (≥ 1114) | 2.9% (1.4-4.4%) | 13.5% (10.3-16.6%) | 27.3% (22.9-31.4%) |
| **RSF-selected model** |  |  |  |
| Very low risk (< 1086) | 0.2% (0.0-0.7%) | 1.6% (0.4-2.8%) | 2.1% (0.7-3.5%) |
| Low risk (1086-1152) | 0.4% (0.0-0.9%) | 1.0% (0.1-1.9%) | 1.5% (0.4-2.5%) |
| Medium risk (1152-1220) | 1.0% (0.1-1.9%) | 2.8% (1.3-4.3%) | 9.2% (6.4-12.0%) |
| High risk (≥ 1220) | 3.1% (1.6-4.7%) | 13.2% (10.0-16.2%) | 25.8% (21.6-29.9%) |
| **ELSA dataset** |  |  |  |
| **LASSO-selected model** |  |  |  |
| Very low risk (< 985) | 0.0% | 0.4% (0.0-1.2%) | 0.8% (0.0-2.0%) |
| Low risk (985-1046) | 0.0% | 0.9% (0.0-1.8%) | 3.6% (1.5-5.7%) |
| Medium risk (1046-1114) | 1.3% (0.4-2.2%) | 3.9% (2.3-5.4%) | 8.0% (5.5-10.4%) |
| High risk (≥ 1114) | 9.8% (7.7-11.7%) | 15.9% (13.4-18.4%) | 23.8% (20.5-26.9%) |
| **RSF-selected model** |  |  |  |
| Very low risk (< 1086) | 0.0% | 0.5% (0.0-1.4%) | 0.5% (0.0-1.4%) |
| Low risk (1086-1152) | 0.0% | 0.2% (0.0-0.7%) | 2.1% (0.5-3.7%) |
| Medium risk (1152-1220) | 1.4% (0.4-2.3%) | 3.6% (2.0-5.1%) | 7.4% (4.9-9.8%) |
| High risk (≥ 1220) | 8.6% (6.8-10.3%) | 14.7% (12.4-16.9%) | 22.5% (19.5-25.4%) |

Risk group categories were defined based on quartiles of the risk score distribution in the WHII Training set; “Events” indicates the number of incident diabetes cases; Each cut-off value was assigned to the group with the higher score range; confidence intervals were not estimable when no events occurred.

**Table S18 Variance inflation factors for the best subset-selected Cox model in the WHII Training set**

| **Predictor** | **VIF** |
| --- | --- |
| **Ethnicity** | 1.10 |
| **Sex** | 1.36 |
| **WHtR** | 1.28 |
| **TyG index** | 1.59 |
| **Fasting glucose** | 1.16 |
| **Family history of diabetes** | 1.06 |
| **CVD medication** | 1.08 |
| **Smoking** |  |
| Ex-smoker | 1.23 |
| Current smoker | 1.25 |
| **Age** | 1.10 |
| **Physical activity** |  |
| Moderate | 1.89 |
| Active | 1.97 |
| **DBP** | 1.18 |
| **HDL-C** | 1.53 |

VIF, variance inflation factor; WHtR, waist-to-height ratio; HDL-C, high-density lipoprotein cholesterol; TyG index, triglyceride-glucose index; DBP, diastolic blood pressure; CVD, cardiovascular disease.

TyG index = ln(fasting triglycerides [mg/dL] × fasting plasma glucose [mg/dL]/2).

**Table S19 Assessment of non-linearity for continuous predictors in the best subset-selected model in the WHII Training set**

| **Predictor** | **P value for non-linearity** |
| --- | --- |
| **Age** | 0.152 |
| **Fasting glucose** | 0.035 |
| **TyG index** | 0.233 |
| **DBP** | 0.244 |
| **HDL-C** | 0.893 |
| **WHtR** | 0.271 |

WHII, Whitehall II; WHtR, waist-to-height ratio; HDL-C, high-density lipoprotein cholesterol; TyG index, triglyceride-glucose index; DBP, diastolic blood pressure.

TyG index = ln(fasting triglycerides [mg/dL] × fasting plasma glucose [mg/dL]/2).

**Table S20 Comparison of linear and restricted cubic spline specifications for** **fasting glucose in the best subset-selected model in the WHII Test set**

| **Time** | **Linear C-index**  **(95% CI)** | **RCS C-index**  **(95% CI)** | **C-index difference**  **(95% CI)** | **Linear O/E ratio**  **(95% CI)** | **RCS O/E ratio**  **(95% CI)** |
| --- | --- | --- | --- | --- | --- |
| 5 years | 0.747 (0.637, 0.841) | 0.748 (0.641, 0.841) | 0.001 (-0.008, 0.010) | 0.852 (0.551, 1.206) | 0.857 (0.553, 1.213) |
| 10 years | 0.788 (0.731, 0.841) | 0.791 (0.735, 0.843) | 0.003 (-0.002, 0.008) | 1.050 (0.862, 1.254) | 1.055 (0.867, 1.258) |
| 15 years | 0.802 (0.769, 0.836) | 0.802 (0.769, 0.836) | 0.000 (-0.003, 0.003) | 0.935 (0.816, 1.057) | 0.937 (0.818, 1.058) |

Fasting glucose was modelled as either a linear term or using restricted cubic splines with four knots. C-index differences were calculated as RCS minus the linear specification.

WHII, Whitehall II; CI, confidence interval; O/E, observed-to-expected; RCS, restricted cubic spline.

The 95% confidence intervals were obtained using 1000 bootstrap samples.

In the WHII Training set, evidence of non-linearity was observed for fasting glucose in the best subset-selected model (P for non-linearity = 0.035). The AIC was 8263.866 when fasting glucose was modelled as a linear term and 8261.711 when fasting glucose was modelled using restricted cubic splines. In the WHII Test set, absolute differences in C-index between the linear and RCS specifications for fasting glucose were no greater than 0.003 at 5, 10, or 15 years, and the 95% confidence intervals for these differences all included zero. O/E ratios were also nearly identical between the two specifications. Fasting glucose was therefore retained as a linear term to maintain model parsimony and facilitate implementation.

**Table S21 Risk score, risk-group definitions, and discrimination of the prediction tool based on the best subset-selected model**

| **Component** | **WHII Training set** | **WHII Test set** | **ELSA dataset** |
| --- | --- | --- | --- |
| **Risk score formula** | Risk Score = 62 × sex (1 if female, otherwise 0) + 97 × non-white (1 if non-white, otherwise 0) + 1 × age (year) + 59 × current smoker (1 if yes, otherwise 0) + 8 × ex-smoker (1 if yes, otherwise 0) + 35 × moderate physical activity (1 if yes, otherwise 0) + 7 × active physical activity (1 if yes, otherwise 0) + 1 × DBP (mmHg) + 67 × family history of diabetes (1 if yes, otherwise 0) + 22 × CVD medication (1 if yes, otherwise 0) + 620 × WHtR + 54 × TyG index − 44 × HDL-C (mmol/L) + 83 × fasting glucose (mmol/L) | | |
| **Risk-group definition** | Very low (< 1245); Low (1245-1311); Medium (1311-1384); High (≥ 1384). | | |
| **C-index for continuous risk score (95% CI)** | 0.768 (0.747, 0.790) | 0.776 (0.744, 0.809) | 0.788 (0.760, 0.817) |
| **C-index for risk groups (95% CI)** | 0.738 (0.717, 0.758) | 0.751 (0.721, 0.780) | 0.716 (0.693, 0.739) |

The risk score formula and risk-group cut-offs were derived in the WHII Training set and applied unchanged to the WHII Test set and ELSA. Each cut-off value was assigned to the group with the higher score range.

WHtR, waist-to-height ratio; HDL-C, high-density lipoprotein cholesterol; TyG index, triglyceride-glucose index; DBP, diastolic blood pressure; CVD, cardiovascular disease.

TyG index = ln(fasting triglycerides [mg/dL] × fasting plasma glucose [mg/dL]/2).

**Table S22** **Discrimination performance of** **LASSO-selected prediction models: C-index and pairwise comparisons (Whitehall II Training set)**

| Model | C index | Model 1 | Model 2 | Model 3 | Model 4 |
| --- | --- | --- | --- | --- | --- |
| Model 1 | 0.701 | - |  |  |  |
| Model 2 | 0.722 | P < 0.001 | - |  |  |
| Model 3 | 0.761 | P < 0.001 | P < 0.001 | - |  |
| Model 4 | 0.765 | P < 0.001 | P < 0.001 | 0.038 | - |

Model 1 (without WHtR and without invasive variables) includes BMI, height, ethnicity, family history of diabetes, smoking, and socioeconomic status;

Model 2 (with WHtR and without invasive variables) includes BMI, height, ethnicity, family history of diabetes, smoking, socioeconomic status, and WHtR;

Model 3 (without WHtR and with invasive variables) includes BMI, height, ethnicity, family history of diabetes, smoking, socioeconomic status, fasting glucose, and TyG index;

Model 4 (the final LASSO-selected prediction model, with WHtR and invasive variables) includes BMI, height, ethnicity, family history of diabetes, smoking, socioeconomic status, WHtR, fasting glucose, and TyG index;

P values are from pairwise comparisons of C-indices using the compareC test (Kang et al., 2015);

BMI, body mass index; WHtR, waist-to-height ratio; TyG index, triglyceride-glucose index;

TyG index = ln(fasting triglycerides [mg/dL] × fasting plasma glucose [mg/dL]/2).

**Table S23** **Discrimination performance of** **RSF-selected prediction models: C-index and pairwise comparisons (Whitehall II Training set)**

| Model | C index | Model 1 | Model 2 | Model 3 | Model 4 |
| --- | --- | --- | --- | --- | --- |
| Model 1 | 0.717 | - |  |  |  |
| Model 2 | 0.729 | P = 0.006 | - |  |  |
| Model 3 | 0.767 | P < 0.001 | P < 0.001 | - |  |
| Model 4 | 0.769 | P < 0.001 | P < 0.001 | 0.051 | - |

RSF, random survival forests;

Model 1 **(**without WHtR and without invasive variables**)** includes age, ethnicity, BMI, height, SBP, DBP, family history of diabetes, and socioeconomic status;

Model 2 **(**with WHtR and without invasive variables**)** includes age, ethnicity, BMI, height, SBP, DBP, family history of diabetes, socioeconomic status, and WHtR;

Model 3 **(**without WHtR and with invasive variables**)** includes age, ethnicity, BMI, height, SBP, DBP, family history of diabetes, socioeconomic status, fasting glucose, TG, HDL-C, and TyG index;

Model 4 (the final RSF-selected prediction model, with WHtR and invasive variables**)** includes age, ethnicity, BMI, height, SBP, DBP, family history of diabetes, socioeconomic status, WHtR, fasting glucose, TG, HDL-C, and TyG index;

P values are from pairwise comparisons of C-indices using the compareC test (Kang et al., 2015);

BMI, body mass index; WHtR, waist-to-height ratio; TG, triglycerides; LDL-C, low-density lipoprotein cholesterol; HDL-C, high-density lipoprotein cholesterol; TyG index, triglyceride-glucose index; SBP, systolic blood pressure; DBP, diastolic blood pressure;

TyG index = ln(fasting triglycerides [mg/dL] × fasting plasma glucose [mg/dL]/2).

**Figure S1 Out-of-bag (OOB) prediction error plot and random forest variable importance plot (Whitehall II Training set)**

The OOB prediction error plot shows that as the number of decision trees increases, the error stabilises, indicating that the model performance has converged.

The variable importance plot shows the relative importance of each predictor in the RSF model. Predictors with higher scores contribute more to model discrimination and help identify key variables for further analysis.

The dashed vertical line indicates the variable selection threshold, defined based on the importance of a noise variable in the random forest. Predictors with importance values above this threshold were retained for further modelling, whereas predictors below the threshold were not selected.

RSF, random survival forests; BMI, body mass index; WHtR, waist-to-height ratio; CVD, cardiovascular disease; TG, triglycerides; LDL refers to low-density lipoprotein cholesterol; HDL refers to high-density lipoprotein cholesterol; TyG refers to triglyceride-glucose index; Glucose refers to fasting plasma glucose; SBP, systolic blood pressure; DBP, diastolic blood pressure; DM.FHx, family history of diabetes; PA, physical activity; SES, socioeconomic status.

TyG index = ln(fasting triglycerides [mg/dL] × fasting plasma glucose [mg/dL]/2).

**Figure S2 Time-dependent concordance index curves for three** **candidate prediction models in Whitehall II**

A. In WHII Training set

B. In WHII Test set

Cox1, full model (21 variables) includes age, sex, ethnicity, height, BMI, WHtR, SBP, DBP, TG, LDL-C, HDL-C, TyG index, fasting glucose, family history of diabetes, smoking status, drinking, fruit/vegetable consumption, physical activity, socioeconomic status, baseline CVD diagnosis, and CVD medication.

Cox2, candidate model for LASSO selection (9 variables) includes ethnicity, WHtR, height, TyG index, fasting glucose, family history of diabetes, smoking, BMI, and socioeconomic status.

Cox3, candidate model for random survival forests selection (13 variables) includes age, ethnicity, BMI, height, SBP, DBP, family history of diabetes, socioeconomic status, WHtR, TG, HDL-C, fasting glucose, and TyG index.

Cox4, candidate model for best subset selection (12 variables) includes age, sex, ethnicity, smoking, physical activity, DBP, family history of diabetes, CVD medication, WHtR, fasting glucose, TyG index, and HDL-C.

RSF, random survival forests; BMI, body mass index; WHtR, waist-to-height ratio; TG, triglycerides; LDL-C, low-density lipoprotein cholesterol; HDL-C, high-density lipoprotein cholesterol; TyG index, triglyceride-glucose index; SBP, systolic blood pressure; DBP, diastolic blood pressure; CVD, cardiovascular disease;

TyG index = ln(fasting triglycerides [mg/dL] × fasting plasma glucose [mg/dL]/2).

**Figure S3 Calibration plots for 5-, 10-, and 15-year diabetes-free survival following the simplified model after bootstrapping (Whitehall II)**

A. LASSO-Cox
a. WHII Training set (5 years)

b. WHII Training set (10 years)

c. WHII Training set (15 years)

d. WHII Test set (5 years)

e. WHII Test set (10 years)

f. WHII Test set (15 years)

Simplified model for LASSO selection (9 variables) includes ethnicity, WHtR, height, TyG index, fasting glucose, family history of diabetes, smoking, BMI, and socioeconomic status.

B. Random survival forests
a. WHII Training set (5 years)

b. WHII Training set (10 years)

c. WHII Training set (15 years)

d. WHII Test set (5 years)

e. WHII Test set (10 years)

f. WHII Test set (15 years)

Simplified model for random survival forests selection (13 variables) includes age, ethnicity, BMI, height, SBP, DBP, family history of diabetes, socioeconomic status, WHtR, TG, HDL-C, fasting glucose, and TyG index.

C. Best subset selection
a. WHII Training set (5 years)

b. WHII Training set (10 years)

c. WHII Training set (15 years)

d. WHII Test set (5 years)

e. WHII Test set (10 years)

f. WHII Test set (15 years)

Simplified model for best subset selection (12 variables) includes age, sex, ethnicity, smoking, physical activity, DBP, family history of diabetes, CVD medication, WHtR, fasting glucose, TyG index, and HDL-C.

BMI, body mass index; WHtR, waist-to-height ratio; TG, triglycerides; HDL-C, high-density lipoprotein cholesterol; TyG index, triglyceride-glucose index; SBP, systolic blood pressure; DBP, diastolic blood pressure; CVD, cardiovascular disease.

TyG index = ln(fasting triglycerides [mg/dL] × fasting plasma glucose [mg/dL]/2).

Note: “Survival” in the calibration plots refers to the probability of remaining free of diabetes (that is, diabetes-free survival).

**Figure S4 Time-dependent concordance index curves for simplified models (internal and external validation sets)**

A. WHII Test set; B. ELSA dataset.

Cox1, simplified model for LASSO selection (9 variables) includes ethnicity, WHtR, height, TyG index, fasting glucose, family history of diabetes, smoking, BMI, and socioeconomic status.

Cox2, simplified model for random survival forests selection (13 variables) includes age, ethnicity, BMI, height, SBP, DBP, family history of diabetes, socioeconomic status, WHtR, TG, HDL-C, fasting glucose, and TyG index.

Cox3, simplified model for best subset selection (12 variables) includes age, sex, ethnicity, smoking, physical activity, DBP, family history of diabetes, CVD medication, WHtR, fasting glucose, TyG index, and HDL-C.

BMI, body mass index; WHtR, waist-to-height ratio; TG, triglycerides; HDL-C, high-density lipoprotein cholesterol; TyG index, triglyceride-glucose index; SBP, systolic blood pressure; DBP, diastolic blood pressure; CVD, cardiovascular disease.

TyG index = ln(fasting triglycerides [mg/dL] × fasting plasma glucose [mg/dL]/2).

**Figure S5 Decision curve analysis for validation and comparison of the candidate simplified models in WHII Test and ELSA datasets**

A. **WHII Test set**
a. 5 years

b. 10 years

c. 15 years

B. **ELSA dataset**
a. 5 years

b. 10 years

c. 15 years

Model LASSO, simplified model for LASSO selection (9 variables) includes ethnicity, WHtR, height, TyG index, fasting glucose, family history of diabetes, smoking, BMI, and socioeconomic status.

Model RSF, simplified model for RSF selection (13 variables) includes age, ethnicity, BMI, height, SBP, DBP, family history of diabetes, socioeconomic status, WHtR, TG, HDL-C, fasting glucose, and TyG index.

Model Best subset, simplified model for best subset selection (12 variables) includes age, sex, ethnicity, smoking, physical activity, DBP, family history of diabetes, CVD medication, WHtR, fasting glucose, TyG index, and HDL-C.

RSF, random survival forests; BMI, body mass index; WHtR, waist-to-height ratio; TG, triglycerides; HDL-C, high-density lipoprotein cholesterol; TyG index, triglyceride-glucose index; SBP, systolic blood pressure; DBP, diastolic blood pressure; CVD, cardiovascular disease;

TyG index = ln(fasting triglycerides [mg/dL] × fasting plasma glucose [mg/dL]/2).

“All” corresponds to treating all individuals as if at high risk, whereas “None” corresponds to treating none.

**Figure S6 Kaplan-Meier curves for incident diabetes by risk group in the WHII Training set, WHII Test set, and ELSA (LASSO-selected model)**

**Panel A:** WHII Training set and WHII Test set

**Panel B**: WHII Training set and ELSA

**Figure S7 Kaplan-Meier curves for incident diabetes by risk group in the WHII Training set, WHII Test set, and ELSA (RSF-selected model)**

**Panel A**: WHII Training set and WHII Test set

**Panel B**: WHII Training set and ELSA

**Figure S8 Nomogram for predicting 5-, 10-, and 15-year diabetes risk (additional analysis for the best subset-selected model)**

WHtR, waist-to-height ratio; HDL, high-density lipoprotein cholesterol; TyG index, triglyceride-glucose index; DBP, diastolic blood pressure; DM.FHx, family history of diabetes; PA, physical activity; CVD, cardiovascular disease; Glucose refers to fasting plasma glucose.

Sex: male = 0, female = 1; Ethnicity: white = 0, non-white = 1; PA: inactive = 0, moderate = 1, active = 2; DM.FHx: no = 0, yes = 1; Smoking: never smoker = 0, ex-smoker = 1, current smoker = 2; CVD medication: no = 0, yes = 1.

Age: year; DBP: mm Hg; fasting glucose: mmol/L; HDL-C: mmol/L;

TyG index = ln(fasting triglycerides [mg/dL] × fasting plasma glucose [mg/dL]/2).
